# Microglia-Derived Exosomal microRNA-151-3p Enhances Functional Healing After Spinal Cord Injury by Attenuating Neuronal Apoptosis *via* Regulating the p53/p21/CDK1 Signaling Pathway

**DOI:** 10.3389/fcell.2021.783017

**Published:** 2022-01-20

**Authors:** Chengjun Li, Tian Qin, Yudong Liu, Haicheng Wen, Jinyun Zhao, Zixiang Luo, Wei Peng, Hongbin Lu, Chunyue Duan, Yong Cao, Jianzhong Hu

**Affiliations:** ^1^ Department of Spine Surgery and Orthopaedics, Xiangya Hospital, Central South University, Changsha, China; ^2^ National Clinical Research Center for Geriatric Disorders, Xiangya Hospital, Central South University, Changsha, China; ^3^ Key Laboratory of Organ Injury, Aging and Regenerative Medicine of Hunan Province, Changsha, China; ^4^ Department of Sports Medicine, Research Centre of Sports Medicine, Xiangya Hospital, Central South University, Changsha, China

**Keywords:** microglia, spinal cord injury, neuronal apoptosis, exosomes, miR-151-3p

## Abstract

Spinal cord injury (SCI) is a catastrophic event mainly involving neuronal apoptosis and axonal disruption, and it causes severe motor and sensory deficits. Due to the complicated pathological process of SCI, there is currently still a lack of effective treatment for SCI. Microglia, a type of immune cell residing in the central nervous system (CNS), need to respond to various stimuli to protect neuronal cells from death. It was also reported that microRNAs (miRNAs) had been identified in microglia-derived exosomes that can be taken up by neurons. However, the kinds of miRNAs in exosome cargo derived from microglia and the underlying mechanisms by which they contribute to neuroprotection after SCI remain unknown. In the present study, a contusive SCI mouse model and *in vitro* experiments were applied to explore the therapeutic effects of microglia-derived exosomes on neuronal apoptosis, axonal regrowth, and functional recovery after SCI. Then, miRNA analysis, rescue experiments, and luciferase activity assays for target genes were performed to confirm the role and underlying mechanism of microglia-derived exosomal miRNAs in SCI. We revealed that microglia-derived exosomes could promote neurological functional recovery by suppressing neuronal apoptosis and promoting axonal regrowth both *in vivo* and *in vitro*. MicroRNA-151-3p is abundant in microglia-derived exosomes and is necessary for mediating the neuroprotective effect of microglia-derived exosomes for SCI repair. Luciferase activity assays reported that P53 was the target gene for miR-151-3p and that p53/p21/CDK1 signaling cascades may be involved in the modulation of neuronal apoptosis and axonal regrowth by microglia-derived exosomal microRNA-151-3p. In conclusion, our data demonstrated that microglia-derived exosomes (microglia-Exos) might be a promising, cell-free approach for the treatment of SCI. MicroRNA-151-3p is the key molecule in microglia-derived exosomes that mediates the neuroprotective effects of SCI treatments.

## Introduction

Spinal cord injury (SCI) is a devastating central nervous traumatic disease that can lead to temporary or permanent neurological deficits (Ahuja CS. et al., 2017; Khorasanizadeh et al., 2019). The incidence of SCI is high, approximately 23.7/million in China (Hao et al., 2021). Over the past decade, a flourishing number of novel strategies have been demonstrated that could promote functional recovery after spinal cord injury in animal models. However, there are no fully restorative treatments for SCI in the clinic (Ahuja C. S. et al., 2017). Most SCI patients are young men and are a main part of the social workforce, and long-term disability imposes a high psychological and financial burden. Therefore, there is a high need to identify a novel effective intervention for repairing injured spinal cord tissue.

Traumatic instigations on the spinal cord typically induce immediate mechanical damage to the spinal parenchyma, along with axonal and cellular damage and hemorrhagic and ischemic formation changes after injury. Neurons exposed to hypoxia result in apoptosis. It seemed that neuronal apoptosis might also contribute to spinal cord cell loss after SCI, inducing further tissue damage. In addition, the sudden interruption of axons after traumatic instigations and limited spontaneous sprouting of axons impede neurological reconstruction, resulting in variable degrees of permanent neurological dysfunction (Tran et al., 2018; Guo M. et al., 2021). Therefore, creating a pro-regeneration microenvironment to protect neurons against apoptosis and enhance neuroplasticity at the axonal level is a novel therapeutic strategy to promote biological repair and functional recovery after SCI.

Immediately following spinal trauma, microglia rapidly accumulate around the injured site of SCI (Bellver-Landete et al., 2019). Microglia, together with blood-derived macrophages, form the main bearer of the immune response in the central nervous system (CNS) and are increasingly recognized to be essential players in the maintenance of normal homeostasis of CNS function. Historically, microglial activation in the injured CNS was generally considered harmful to neurogenesis due to the secretion of proinflammatory cytokines. It was recently proposed that microglia play a beneficial role in SCI repair and that the depletion of microglia inhibits locomotor functional recovery after SCI (Hamzah et al., 2021; Tran et al., 2021; Zhou et al., 2021). Recently, published research also demonstrated that scar-free healing could be achieved after SCI in neonatal mice associated with axon regrowth across the lesion site. However, this positive effect was abolished when microglia were depleted (Li et al., 2020). Microglia could interact with almost all CNS cells during normal status and diseased development by transferring a large number of factors that are crucial for CNS function. One emerging novel propagating model of such cell-to-cell communication is through the release of exosomes (Guo Z. et al., 2021). Microglia-released exosomes can be taken up by neurons and affect recipient cell neurogenesis and neuronal cell apoptosis. Microglia-derived microvesicles have also been reported to regulate neuronal excitability (Guo M. et al., 2021). Microglia-derived exosome cargo contains a variety of factors, including microRNAs (miRNAs), proteins, and mRNAs, and can either target specific cell types or be taken up by neighboring cells (Hou et al., 2020). Among the cargo contents, miRNAs are the most attractive investigated molecules and have been shown to be involved in multiple physiological and pathological processes. A study showed that microglial exosomal miR-124 could inhibit neuronal inflammation and contribute to neurite outgrowth following traumatic brain injury *via* the transfer of exosomes into neurons. Song et al. also demonstrated that type 2 microglia-derived exosomes attenuated ischemic brain injury by promoting neuronal survival through transfer of exosomal miR-124 to neuronal cells (Song et al., 2019). Bioinformatics analysis showed that miR-151-3p was enriched in microglial exosomes (Xu et al., 2019). However, the special role and mechanisms of miR-151-3p from microglia-derived exosomes (microglia-Exos) on the function of neurons and axon growth after SCI require further exploration.

In the current study, we elucidated the therapeutic effect of microglia-Exos on neurological functional recovery after SCI. Exosomes from microglia accumulated in the injured spinal cord and were mainly taken up by neurons. Exosome treatment promotes neurological functional recovery by inhibiting neuronal apoptosis and promoting axon regrowth. miR-151-3p was highly expressed in microglia-Exos and mediated the neuroprotective function of microglia-Exos for SCI repair *in vivo* and *in vitro*. Meanwhile, downstream p53/p21/CDK1 signaling cascade activation was involved in microglia-Exos regulating neuronal apoptosis and axon regrowth. This finding explored a novel underlying mechanism for the application of microglia-Exos for the treatment of SCI.

## Methods

### Animal

Adult female C57BL/6 mice (8 weeks old) were obtained from the animal center at Central South University (Hunan, China). They were housed in a controlled specific pathogen-free (SPF) environment with a light/dark cycle. The animals have free access to food and water. All the procedures applied in this study were approved by the Animal Ethics Committee of Central South University and complied with relevant management regulations.

### Isolation and Identification of Primary Microglia

Neonatal mice (1 day after birth) were used for primary microglial isolation as previously described (Du et al., 2021). Briefly, mice were dissected to isolate the cerebral cortices and carefully peel off the meninges and blood vessels. Then, the brain tissues were cut using microscissors into pieces and digested with enzymes (8 U/ml papain and 125 U/ml DNase, Thermo Fisher Scientific, MA, United States) at 5% CO_2_ and 37°C for 20 min. After the termination of the digestion, the cell suspension was filtered using a 40-μm cell strainer (Corning, NY, United States) and centrifuged at 200 g for 10 min. The cells were resuspended in a T25 culture flask with Dulbecco’s modified Eagle’s medium (DMEM, HyClone, United States) containing 10% fetal bovine serum (FBS, Gibco, United States) and 1% penicillin/streptomycin (Sigma, United States), and the supernatant was removed and washed twice with PBS the next day. On the fourth day, the culture flask was placed at 180 rpm (round per minute) for 30 min on a shaker, and the supernatant was transferred to a poly-d-lysine–coated 12-well plate. After 3 h of incubation, the supernatant was removed to obtain pure microglial cells and examined under an inverted microscope for morphological analysis.

Primary microglial cells were identified with immunocytofluorescence. The cells were fixed in 4% paraformaldehyde and rinsed in PBS. Primary microglia were incubated with primary antibodies against F4/80 and Iba-1 overnight at 4°C. Then, the slide was incubated with secondary antibodies. 4’, 6-diamidino-2-phenylindole (DAPI) was used for nuclear counterstaining. The antibodies used are listed in Supplementary Table S2.

For flow cytometry analysis, the isolated cells were immunolabeled with PerCP-Cy5.5-anti-CD11b antibody, FITC-anti F4/80 antibody, FITC-anti-CD31 antibody, APC-anti-O4 antibody, and PE-anti-GLAST antibody in flow cytometry staining buffer. For IBA-1 staining and analysis, cells were fixed in PBS containing 2% paraformaldehyde, permeabilized with 0.25% Triton X-100 and 1% BSA in PBS, and then immunolabeled with anti-IBA-1 antibody and Alexa Fluor 488-conjugated goat anti-rabbit IgG H&L (Alexa Fluor 488) in flow cytometry staining buffer. A flow cytometric analysis was performed on a BD FACSCanto II cytometer, and the data were analyzed using FlowJo software (FlowJo, LLC, Ashland, OR). The antibodies used are listed in Supplementary Table S2.

### Isolation and Identification of Microglia-Exos

Microglia-Exos were acquired by gradient ultracentrifugation, according to a previous report (Peng et al., 2021). Briefly, microglia cells were cultured in complete medium containing EVs-free FBS for 48 h. The culture medium was centrifuged at 300×g for 10 min, 2000×g for 30 min, and 10,000×g for 30 min to sequentially remove cells, dead cells, and cellular debris. After filtering through a 0.22-μm filter (Millipore, Billerica, United States), the collected supernatant was ultra-centrifuged at 10,000×g for 2 h to completely remove supernatant, and then the exosome pellet was collected by resuspending in PBS. The protein content of exosomes was determined using the Pierce BCA Protein Assay Kit (Thermo Fisher Scientific, United States). The obtained exosomes were stored at −80°C for subsequent experiments.

Microglia cells were transfected with miR-125b-5p inhibitor or negative control (NC) inhibitor (Hanbio, China) at a concentration of 100 nM using Lipofectamine 3000 (Hanbio, China) after cell attachment. The sequences of miR-151-3p and negative control (NC) inhibitor were as follows: miR-151-3p-inhibitor (5′-CCU​CAA​GGA​GCC​UCA​GUC​UAG-3′); miR-151-3p-NC (5′-UCA​CAA​CCU​CCU​AGA​AAG​AGU​AGA-3′). 3 days post transfection, the total RNA from the microglia cells was extracted with TRIzol reagent (Invitrogen, United States) for further analysis.

For exosome identification, the purified exosomes were fixed with glutaraldehyde solution in PBS. After dropping on the copper net and staining with 2% phosphotungstic acid for 2 min, the exosomes were observed by transmission electron microscopy (FEI, United States). The diameters of exosomes were measured using a nanotracking analysis system. In addition, Western blotting was used to detect exosomal biomarkers, including CD9, CD63, and Tsg101.

### Exosomes Labeling and Tracking

For exosome uptake tracking, PKH67 and PKH26 kit (Sigma–Aldrich, San Louis, United States) fluorescence labeling of microglia-Exos was performed according to the instruction manual described. For *in vitro* uptake experiment, 50,000 primary neurons were seeded into a 24-well plate. 24 h later, 10 ug labeled exosomes were added into each well. After 48 h of incubation, neurons were fixed for *in vitro* exosome uptake experiments. For *in vivo* tracking, 200 μg exosomes in 100 ul saline were injected through the tail vein for three consecutive days after SCI. 3 days post injury, exosome-treated mice were terminated, and the spinal segment was harvested and dehydrated for sectioning and immunofluorescence analysis.

### Primary Cortical Neurons Cell Culture

Primary cortical neurons were isolated from fetal mice (E14-16) as previously described (Guo Z. et al., 2021). Briefly, the cortical brains of the fetal mice were removed and placed into a cold PBS solution. Then, the meningeal tissues were carefully wiped off, and the brain tissues were transferred to a 15-ml centrifuge tube containing cold DMEM. They were gently blown upon using a 1-ml pipette for homogenization. After filtration using a 70-µm filter and centrifugation (1,000 rpm, 5 min), the supernatant was removed, and the cells were resuspended in a neurobasal medium (Cyagen, US) containing 2% B27 (Gibco, United States), 1% glutamine (Gibco, United States), and 1% penicillin-streptomycin (Sigma, United States). After cell adhesion (4 h), neurons were treated with myelin debris to simulate the *in vivo* microenvironment after spinal cord injury (SCI) to inhibit axon growth as previously described. 24 h after the neurons were seeded, 10 μg exosomes (exosomes/miR-151-3p^IN^-exosomes) were incubated for 2 days with the cultured neurons for further Western blot, qRT-PCR, and neurite length measurement. The neurons in the control group were treated with the same volume of PBS. The average neurite length was calculated using ImageJ with a plugin of Ridge Detection by dividing the length of all neurites by the number of neurons.

### Myelin Debris Preparation

The myelin debris was obtained following a previous research study (Rolfe et al., 2017). Briefly, after anesthesia, the mice were terminated by cervical dislocation. The brain tissues were harvested and cut into pieces in 0.32 M sucrose solution. Then, the tissues were homogenated by ultrasound, added into 0.83M sucrose solution, and centrifuged at 100,000 g at 4°C for 45 min. The crude myelin debris was homogenized again and resuspended into Tris-Cl buffer for centrifugation at 100,000 g at 4°C for 45 min. We discarded the supernatant and resuspended the myelin debris into sterile PBS for centrifugation at 220,000 g at 4 °C for 10 min. Then, we removed the supernatant, weighed the debris pellet, and suspended it to 100 mg/ml with PBS. Purified myelin debris was stored at −80°C for further experiments.

### Contusive SCI Models and Treatment

A modified Allen’s impact method was used to induce the spinal contusion SCI model as previously described (Li et al., 2021). Briefly, the mice were anesthetized with a ketamine/xylazine mixture (120 mg/3.3 mg/kg) solution. Then, a dorsal median incision was made at thoracic level 10 to expose the spinal cord carefully. After laminectomy at T10, a rod weighing 10 g was dropped onto the spinal cord at a height of 25 mm without creating any unnecessary pressure on the spinal cord using an NYU impactor; then the muscles and skin were sutured sequentially. Once the mice were awake, 1.5 ml (0.006 mg/ml) buprenorphine was applied subcutaneously for the first 24–48 h to relieve the pain caused by the surgery. The bladders should be manually expressed twice a day for the following week until the return of bladder function. For the sham group, the mice underwent laminectomy without establishment of the SCI model. For the treated groups, microglial cell-derived exosomes (200 μg exosome/miR-151-3p^IN^-exosomes) were injected through the tail vein for three consecutive days after SCI establishment. The mice in the control group were treated with the same volume of saline through the same approach.

### Apoptosis Analysis

A TUNEL (TdT-mediated dUTP Nick End Labeling) apoptosis detection kit (Cy3, Beyotime, China) was applied to detect apoptotic cells in spinal sections. Slices were selected near the central canal horizontally. After fixation and permeation, the sections were incubated with TUNEL solution for 1 h at room temperature. Then, the sections were washed 3 times with PBS and stained with DAPI. The region of interest (ROI) was selected from the anterior horn of the spinal cord at the 500 μm rostrally adjacent to the injured epicenter. Apoptotic cells were observed using a fluorescence microscope (Zeiss, Oberkochen, Germany). ImageJ was used to count the total cells and TUNEL-positive cell numbers. The apoptosis of neurons in different treatment groups was measured using an Annexin V (FITC)/propidium iodide (PI) detection kit (BD Biosciences, United States) and analyzed using a flow cytometer (BD-LSRII, United States) as previously described (Zhang et al., 2016).

### qRT–PCR

Total RNA was extracted from microglial cells (3 days post transfection), neurons (3 days post exosomal treatment), and microglia-Exos by using TRIzol reagent (Invitrogen, United States) and reverse transcribed to complementary DNA (cDNA) using a PrimeScript™ RT Reagent kit (Promega, United States) based on the manufacturer’s instructions. Reverse transcription of microRNAs was performed using an miRNA First Strand cDNA Synthesis kit (Tailing Reaction, Sangon Biotech, China). The expression of targeted genes or microRNAs was measured using a GoTaq qPCR Master Mix kit (Promega, United States) and a quantitative PCR system (ABI, United States). GAPDH served as the internal reference of P53, and U6 was the internal reference of microRNAs. The relative quantitative expression was analyzed using the 2 ^(−ΔΔCt)^ method. The primers used are listed in Supplementary Table S1.

### Western Blot

Total protein of cultured neurons (3 days post exosomal treatment) and microglia-Exos was extracted by using RIPA lysis buffer (Beyotime, China), and the protein concentration was detected using a BCA kit. The denatured proteins were separated by 10% SDS–PAGE and then electrophoretically transferred to PVDF membranes (Millipore) according to previously described methods. After blocking with 5% fat-free milk, the membranes were incubated at 4°C overnight with primary antibodies against CD9, CD63, Tsg101, p53, p21, CDK1, Bcl-2, bax, cleaved-caspase3, and β-actin. The membranes were washed with 1% TBST 3 times and incubated with HRP-labeled secondary antibody at room temperature for 90 min. Enhanced chemiluminescence reagent (ECL) was used to visualize immunoreactive bands. β-actin was used as an internal control. The antibodies used in this study are listed in Supplementary Table S2, including the catalog numbers and indicated dilutions.

### Histology and Immunofluorescence Analysis

After being anesthetized with ketamine/xylazine mixture (120 mg/3.3 mg/kg) solution, mice were perfused with saline *via* the left ventricle to allow rapid and sufficient draining of blood flow, followed by 10% formalin solution for fixation. After perfusing, a 10-mm spinal segment including the injury epicenter was harvested and dehydrated for sectioning. For hematoxylin-eosin (HE) staining, the spinal segment was embedded in paraffin, horizontally sectioned at a thickness of approximately 8 μm, and stained according to the instructions of the HE staining kit (Solarbio, China). Images were captured using an Olympus photomicroscope (magnification, ×400). The cavity area of HE staining was outlined manually and calculated using ImageJ.

For immunofluorescence staining, the spinal segment was embedded in OCT compound (Takara, United States) and horizontally cut into 16-µm slices. The slices were incubated overnight with primary antibodies, including anti-MAP2, anti–5-HT, and anti-NeuN. After washing with PBS, the slices were then incubated with secondary antibodies. 4′,6-Diamidino-2-phenylindole, dilactate (DAPI; 1 μg/ml, Thermo Fisher Scientific) was used for nuclear counterstaining. The region of interest (ROI) of MAP2 was selected from the anterior horn of the spinal cord at the 500 μm rostrally adjacent to the injured epicenter. Images were captured using an Axio Imager one microscope (Zeiss, Oberkochen, Germany). The antibodies used in this study are in Supplementary Table S2.

### Fluorescent miRNA *in situ* Hybridization

Biotin-labeled miR-151-3p (Sangon Biotech, China) was constructed for fluorescent miRNA *in situ* hybridization as previously described (Guo M. et al., 2021). Briefly, formalin-fixed paraffin-embedded sections were cut into 8-µm thick sections. The slices were dewaxed with xylene and rehydrated in an ethanol gradient. Then, the slices were treated with proteinase K (20 μg/ml, Ambion, United States), followed by prehybridization for 1 h at 37°C. Then, the prehybridization buffer was removed and incubated with the miR-151-3p-biotin probe (sequence: 5′-biotin-CCUCAAGGAGCCUCAGUCUAG-biotin-3′) at 60°C. After overnight incubation, the slices were washed with 2 × SSC, 1 × SSC, and 0.5 × SSC. After blocking for 1 h at room temperature with blocking buffer (3% BSA in 0.1% PBST), the slices were incubated with Cy3-conjugated streptavidin (Jackson ImmunoResearch, United States) for 1 h. Anti-NeuN antibody was used to stain the nuclei of neurons, followed by the corresponding secondary antibody. DAPI was used to label the cell nucleus. The region of interest (ROI) was selected from the anterior horn of the spinal cord at the 500 μm rostrally adjacent to the injured epicenter in the horizontal sections. For quantification, three sections from different animals in each group were chosen to evaluate the relative immunofluorescent intensity ratio of miR-151-3p/Neun using ImageJ.

### Luciferase Reporter Assay

The p53 plasmid and mmu-miR-151-3p mimic were constructed by Hanbio Co., China. Briefly, wild-type 3′UTR of Trp53 containing the mmu-miR-151-3p binding site and the mutated binding sequence were cloned into pSI-Check2 plasmids (Hanbio Co., China), referred to as Trp53-WT and Trp53-MUT, respectively. A human renal epithelial cell line (293T cells) was cotransfected with 3′UTR luciferase reporter constructs (3′UTR- Trp53-wild type, 3′UTR- Trp53-mutant) and miRNAs (miR-151-3p-negative control (NC) or miR-151-3p-mimic). After 2 days of transfection, a Dual-Luciferase Reporter Gene Assay Kit (Beyotime, China) was used to detect the binding relationship between Trp53 and miR-151-3p based on the instructions. The luciferase activity of each group was measured using a microplate reader. The sequence of the miR-151-3p mimic was 5′ CUA​GAC​UGA​GGC​UCC​UUG​AGG3’. The sequence of miR-151-3p-NC is 5′UCA​CAA​CCU​CCU​AGA​AAG​AGU​AGA3’.

### Locomotor and Sensory Function Assessment

The BMS scoring system was applied to evaluate the locomotor function as previously described (Basso et al., 2006). Two well-trained investigators who were blinded to treatment assessed hindlimb movements. The BMS score ranges from 0 to 9 (0 represents complete paralysis, while nine indicates normal mobility). A thermal paw stimulation apparatus (IITC, United States) was used for thermal sensory function assessment. When the mice felt pain and withdrew their paw, the reaction time was recorded as the withdrawal threshold. The experiment was repeated 3 times with an interval of 10 min, and the average withdrawal threshold was taken as the final result. The locomotor and sensory function was assessed at 1, 3, 7, 14, 21, 28, 35, 42, and 56 days post injury.

### Neuroelectrophysiology Analysis

Motor evoked potential (MEP) and latent period were used to assess neurological connectivity as previously described 56 days post-injury after treatment (Schlag et al., 2001). After the mice were anesthetized, we exposed the bregma of mice and used a stereotaxic device for positioning. Stimulating electrodes were placed on the surface of the skull at the point of 1 mm caudal, 0.5 mm lateral to the bregma and −4 mm caudal, 0.6 mm lateral to the bregma, separately. Recording electrodes were inserted into the tibialis anterior muscle of the contralateral hindlimb. Subcutaneous tissue between the stimulating and recording electrodes was used to insert the reference electrode. Electrical stimulation was performed at 3 V voltage and 333 Hz and repeated every 2 seconds. The MEP was calculated by summing the voltage values of the peak and the trough. The potential period was measured from the initiation time of the first response point to the wave starting to change drastically.

### Statistical Analysis

We analyzed the data using GraphPad Prism 8 software. A *p* value <0.05 indicates statistical significance. Data were shown as the mean ± standard deviations (SDs). Statistical comparisons of two groups were conducted using a two-tailed unpaired Student’s *t*-test. Differences among multiple groups were analyzed with one-way ANOVA followed by Tukey’s *post hoc* tests. The analysis of BMS scores and tactile sensory tests were analyzed with repeated-measures two-way ANOVA at different time points.

## Results

### The Identification of Microglia and Microglia-Exos

**FIGURE 1:**
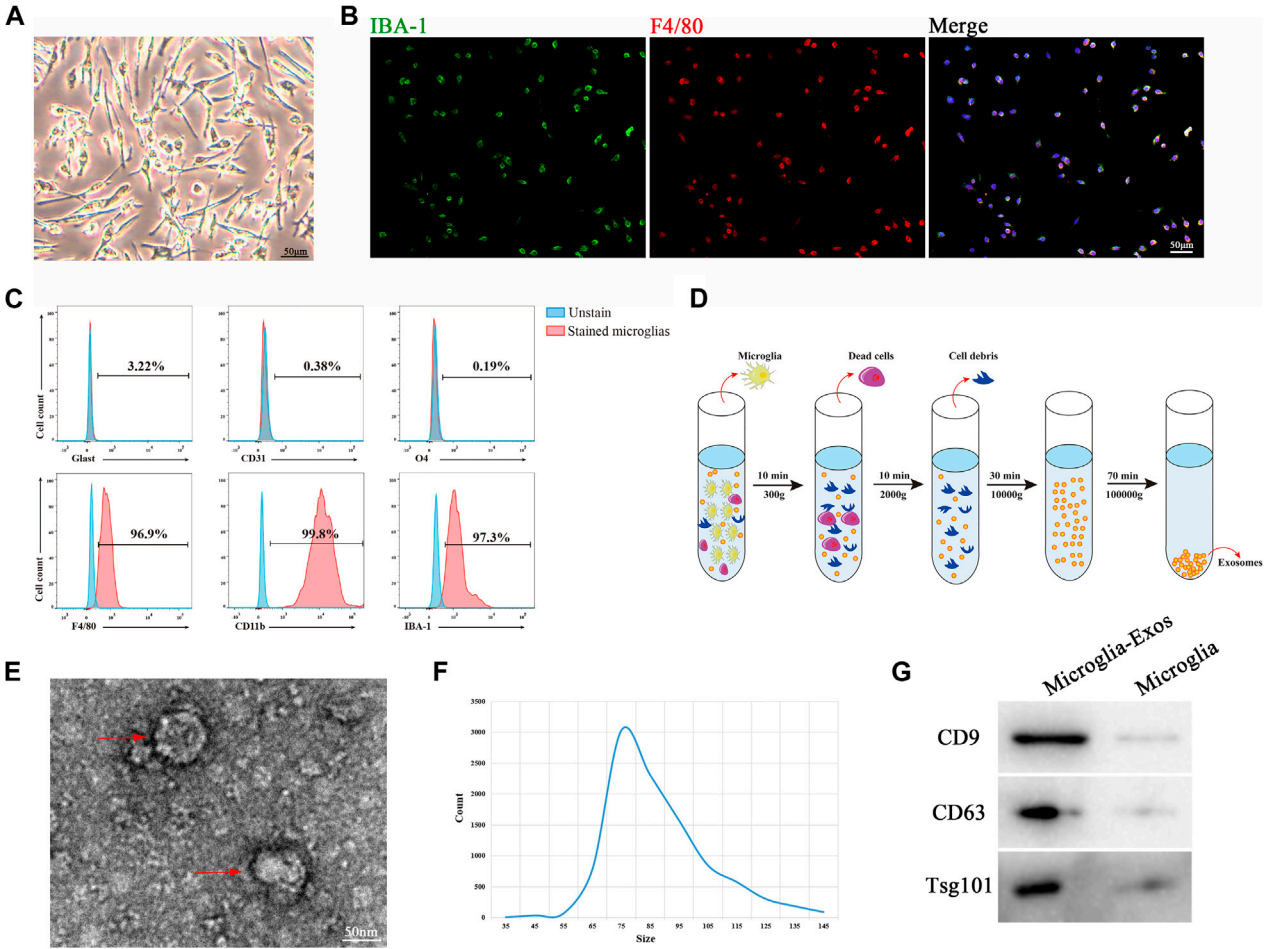
Morphology and characterization of microglia cells and microglia-Exos. **(A)** Representative microscopic image of microglia cells. Scale bar: 50 μm. **(B)** Representative fluorescent images of microglia cells staining with Iba-1 (green) and F4/80 (red). Scale bar: 50 μm. **(C)** Flow cytometry analysis of microglia-positive markers F4/80, CD11b, and IBA-1 and -negative markers Glast, CD31, and O4. **(D)** Schematic depiction of super-centrifugation used to isolate and purify exosomes. **(E)** Transmission electron microscopy (TEM) image of microglia cell–derived exosomes. Scale bar: 50 nm. **(F)** Nanotracking analysis (NTA) showed that the diameter of microglia-derived exosomes ranged from 35 to 145 nm. **(G)** Western blot analysis of exosome markers CD9, CD63, and Tsg101.

Primary microglial cells were isolated from the cerebral cortex of neonatal mice (1 day post birth) and showed a long spindle shape (Figure 1A). Immunocytochemistry demonstrated that the cells expressed the microglial markers IBA-1 and F4/80 (Figure 1B). To further identify the purity of isolated microglia, we used flow cytometry with the microglia positive markers F4/80, IBA-1, and CD11b and the negative markers glast, CD31, and O4 to exclude astrocytes, endothelial cells, and oligodendrocytes separately, as depicted in Figure 1C. These results confirmed the phenotypic characterization of primary microglia. Exosomes were isolated from microglial cell culture medium by ultracentrifugation (Figure 1D) and identified using TEM, nanoparticle tracking analysis (NTA), and Western blot analysis. As shown in Figure 1E, TEM images showed that microglia-Exos had a typical cup-shaped morphology. The diameter of the particles was in the range of 35–145 nm, which was further validated by nanotracking analysis (Figure 1F). Western blot analysis indicated that microglia-Exos expressed the exosomal markers CD9, CD63, and Tsg101 in isolated exosomes (Figure 1G).

### Microglia-Exos Improve Functional Healing After SCI

**FIGURE 2:**
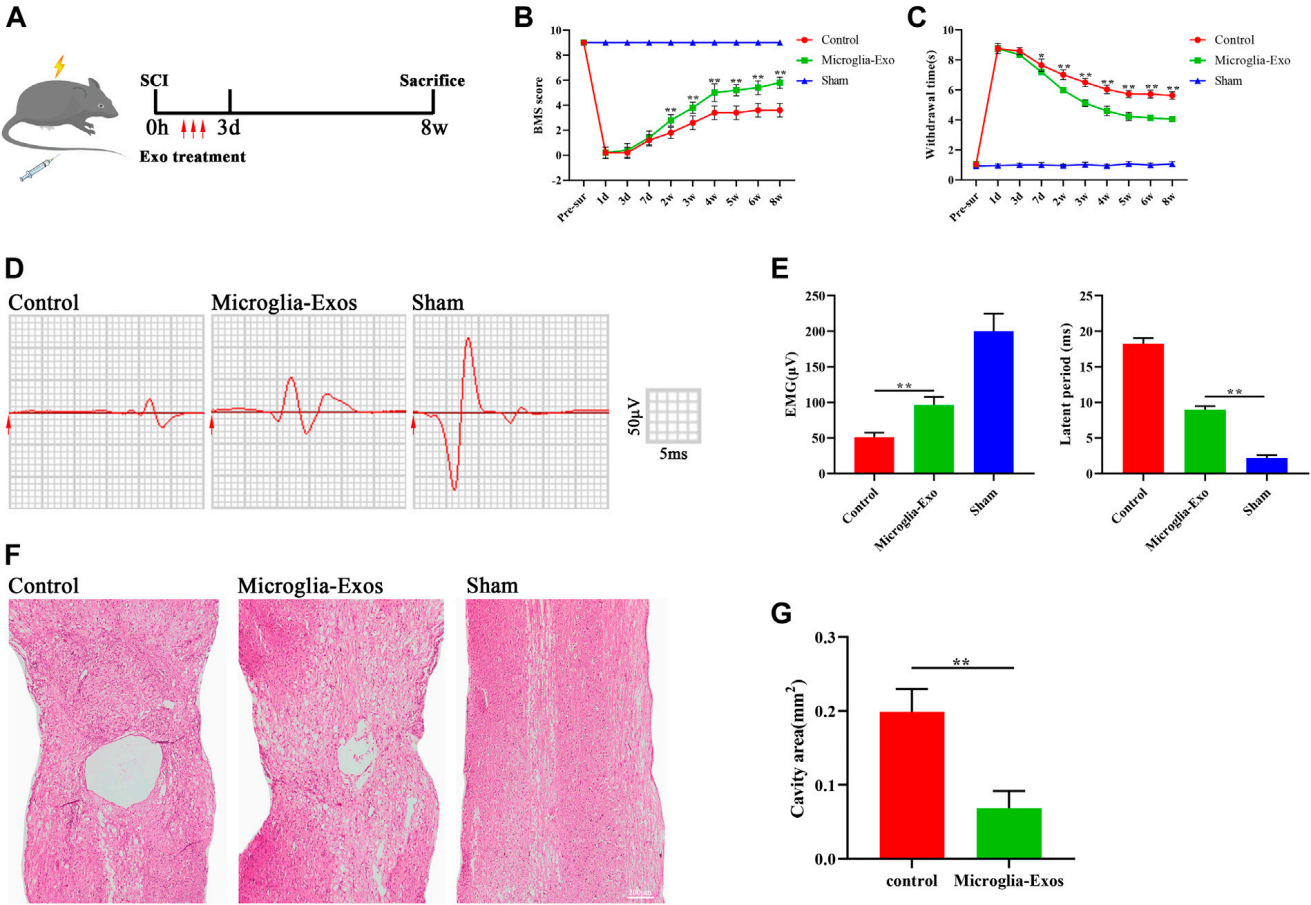
Microglia-Exos improved neurological function after SCI**. (A)** Schematic depiction of experiment procedures. **(B)** BMS scores in sham, control, and microglia-Exo–treated groups at different time points post-SCI. N = 5 per group. **(C)** Hind paw withdrawal times of sham, control, and microglia-Exo–treated groups at different time points post-SCI. N = 5 per group. **(D)** Neuroelectrophysiology analysis of sham, control, and microglia-Exo–treated groups at 56 days post-SCI. **(E)** Quantification of motor evoked potential (MEP) and latent period in **(D)**. N = 5 per group. **(F)** Representative images of hematoxylin-eosin (HE) staining of sham, control, and Microglia-Exo–treated groups. Scale bar: 200 μm. **(G)** Quantification of cavity area of **(F)**. N = 5 per group. Data are presented as the mean ± SD. *p < 0.05, **p < 0.01.

To investigate the effect of Microglia-Exos on functional healing after SCI, we labeled Microglia-Exos with PKH26 and injected them *via* the tail vein for three consecutive days after injury (Figure 2A). Microglia-Exo–treated mice exhibited improved locomotor function recovery compared with the control group 2 weeks after injury until the end of observation, as shown by BMS scores (Figure 2B). In addition, as shown in Figure 2C, better sensory function recovery was observed in the microglia-Exo–treated group from 7 days after injury to the end of the experiment than in the control groups. In addition, we performed neuro-electrophysiology analysis to evaluate the neurological connectivity of the spinal cord to the hindlimb muscles at 56 days after injury among different treatment groups. As depicted in Figure 2D and Figure 2E, mice treated with microglia-Exos showed a higher motor evoked potential (MEP) and shorter latent period than those in the control group, indicating that microglia-Exos could promote nerve conduction capacity after SCI. The cavity area of the spinal cord in hematoxylin-eosin (HE)-stained sections was smaller in the microglia-Exo–treated group than in the control group (Figures 2F,G). Collectively, microglia-Exo treatment reduced the injured size of the cavity and promoted locomotor and sensory function recovery after spinal cord injury.

### Microglia-Exos Enhance Axon Growth and Inhibit Neuronal Apoptosis *via* the p53/p21/CDK1 Pathway

**FIGURE 3:**
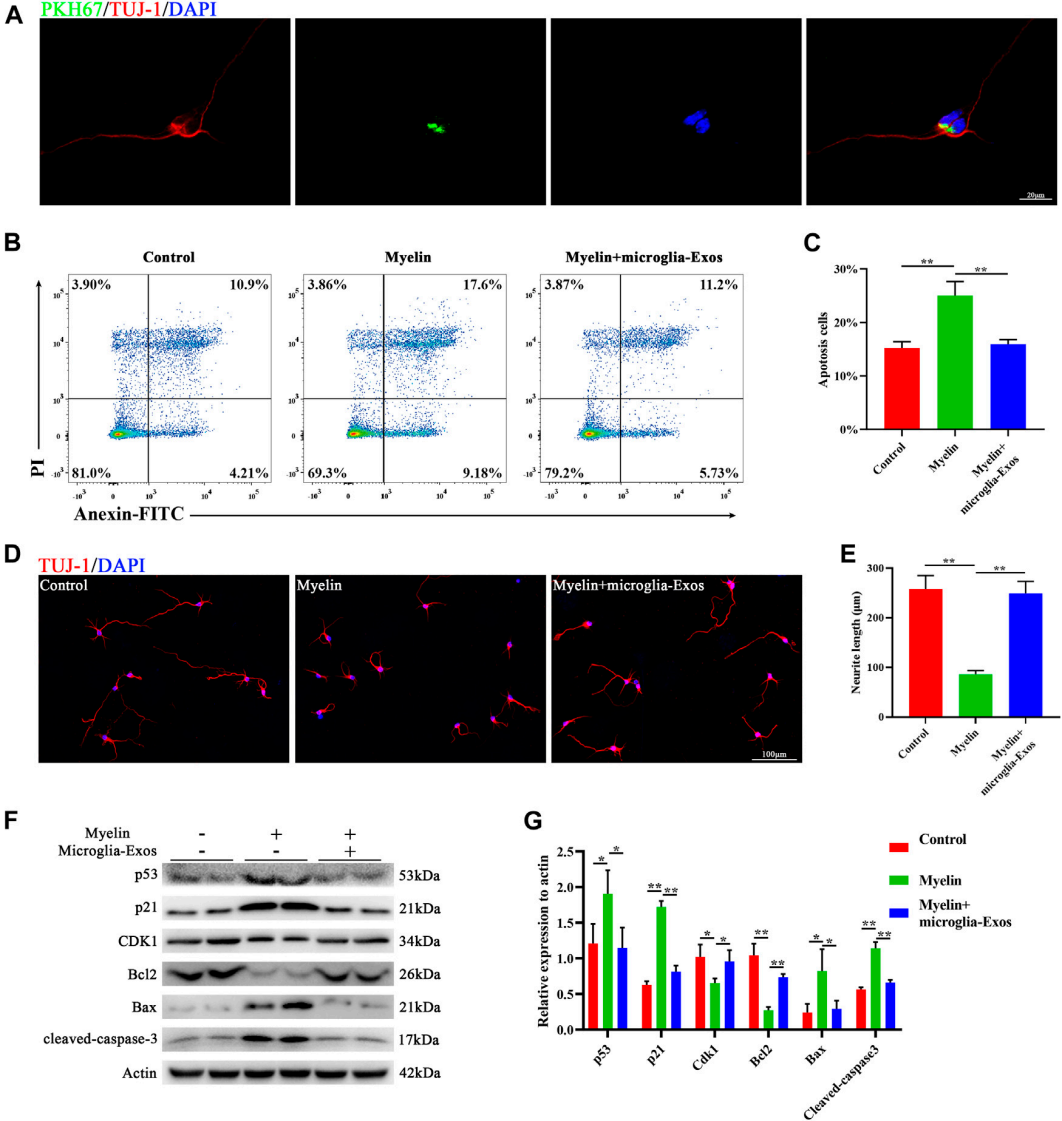
Microglia-Exos enhance neurite growth and inhibit neuronal apoptosis and suppress the p53/p21/CDK1 pathway**. (A)** Representative image of exosomes (PKH67, green) taken up by neurons (Tuj-1, red). Scale bar: 20 μm. **(B)** Flow cytometry of Annexin/PI staining in control, myelin debris, and myelin debris + microglia-Exo–treated groups. **(C)** Quantification of the apoptosis rate of neurons in **(B)**. N = 3 per group. **(D)** Representative fluorescent images of neurons staining with Tuj-1 (red) of control, myelin debris, and myelin debris + Microglia-Exo–treated groups. Scale bar: 100 μm. **(E)** Quantification of neurite length in **(D)**. N = 5 per group. **(F)** Western blot of p53, p21, CDK1, apoptosis-related protein Bax, and cleaved-caspase-3 and anti-apoptosis protein Bcl2 in control, myelin debris, and myelin debris + microglia-Exo–treated groups. **(G)** Quantification of relative protein expression levels in **(F)**. N = 3 per group. Data are presented as the mean ± SD. *p < 0.05, **p < 0.01.

To explore the underlying mechanism of microglia-Exos on neurological functional recovery, we labeled microglia-Exos with PKH67 (Sigma, green) and added them to the neuron culture medium. As shown in Figure 3A, exosomes could be taken up by neurons (stained by Tuj-1). Myelin debris was one of the key factors leading to the inhibitory microenvironment for axon growth and neuronal apoptosis (Sosa et al., 2015; Wu et al., 2021). Here, we used purified myelin debris to simulate the inhibitory microenvironment *in vivo* after SCI. Myelin debris treatment increased neuronal apoptosis using Annexin/PI staining, and this effect was reversed after microglia-Exo treatment (Figures 3B,C). Western blot analysis also indicated that neuronal apoptosis was induced by myelin debris treatment, accompanied by increased levels of the apoptosis-related proteins Bax and cleaved-caspase-3 and decreased expression of the antiapoptotic protein Bcl2 (Figures 3F,G). Myelin debris inhibited cortical neuron axon regrowth. However, the decrease in axon regrowth with the administration of myelin debris was markedly blocked by the addition of microglia-Exos (Figures 3D,E). p53 has been reported to mediate cell apoptosis in CNS injury (Hong et al., 2010). The p53-p21 pathway induced by DNA damage could inhibit cyclin-dependent kinase (CDK) activities, thus resulting in cell apoptosis (Karimian et al., 2016). We measured the protein levels of the p53/p21/CDK1 signaling pathway by Western blotting. Myelin debris treatment increased the expression levels of p53 and p21 while decreasing cyclin-dependent kinase 1 (CDK1) in neurons, while this effect was attenuated when cells were treated with microglia-Exos (Figures 3F,G). To investigate whether exosomes affect neuronal apoptosis and axon growth through the p53/p21 signaling pathway, we used Aaptamine (MedChemExpress, US) at a concentration of 30 μm to simulate p53. Aaptamine is an alkaloid isolated from sponge aaptos, which is a competitive antagonist of α-adrenergic receptors to activate the P21 promoter independently of the p53 pathway (Aoki et al., 2006). As shown in Supplementary Figure S2A,B, compared to the microglia-Exo–treated group, expression level of p21 and apoptosis-related proteins Bax and cleaved-caspase-3 in neurons was increased, while expression of CDK1 and antiapoptotic protein Bcl2 was decreased in the microglia-Exos + Aaptamine–treated group. In Supplementary Figure S2C,D of Annexin/PI staining and Supplementary Figure S2E,F of Tuj-1 staining, neurons in the microglia-Exos + Aaptamine–treated group showed an increased apoptosis cell rate and decreased axon regrowth compared to the microglia-Exo–treated group. These results indicated that microglia-Exos can enhance axon growth and inhibit neuronal apoptosis by suppressing the apoptosis-related p53/p21/CDK1 pathway.

**FIGURE 4:**
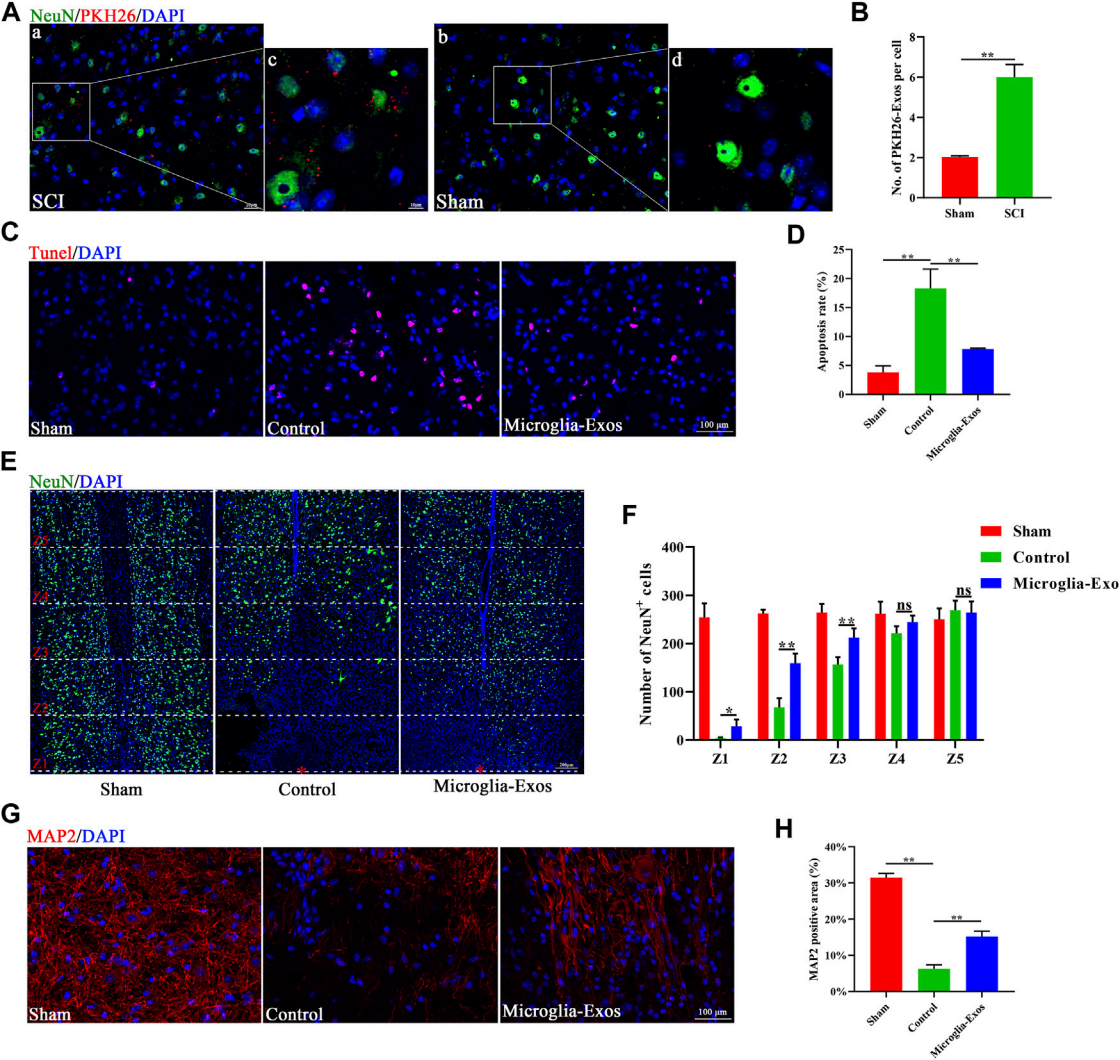
Microglia-Exos lessen neuron loss and facilitate axon growth by suppressing neuron apoptosis**. (A)** Representative images of exosomes (PKH26, red) uptaken by neurons in sham and SCI groups. Scale bar: a, b (20 μm) and c, d (10 μm). **(B)** Quantification of number of PKH26-exos per cell in **(A)**. N = 3 per group. **(C)** Representative images of TUNEL staining of sham, control, and Microglia-Exo–treated groups. Scale bar: 100 μm. **(D)** Quantification of the apoptosis rate in **(C)**. N = 5 per group. **(E)** Representative staining images of NeuN (green) of sham, control, and Microglia-Exo–treated groups at different zones. The red star indicates the epicenter of the injury site. Scale bar: 200 μm. **(F)** Quantification of number of NeuN-positive cells at different zones in **(E)**. N = 5 per group. **(G)** Representative staining images of MAP2 (red) of sham, control, and Microglia-Exo–treated groups. Scale bar: 100 μm. **(H)** Quantification of the MAP2-positive area in **(G)**. N = 5 per group. Data are presented as the mean ± SD. *p < 0.05, **p < 0.01, ns = not significant.

To further explore the underlying mechanism *in vivo*, we injected PKH26-labeled exosomes through the tail vein after SCI. Fluorescence images of frozen spinal sections showed that PKH26-labeled exosomes mainly accumulated in neurons after SCI. The neurons in the injured spinal cord took up more microglia-Exos than those in the sham group (Figures 4A,B). As shown in Figure 4C, the apoptosis rate was markedly increased after SCI but decreased after microglia-Exos administration, as shown by TUNEL staining (Figures 4C,D). SCI usually causes neuronal loss, which is related to neuronal apoptosis. We sequentially counted the NeuN^+^ cells in areas of 0–500 μm, 500–1,000 μm, 1,000–1,500 μm, 1,500–2000 μm, and 2000–2,500 μm roaster from the epicenter. Compared to the sham group, the number of NeuN^+^ cells decreased after injury but was ameliorated after microglia-Exo treatment (Figures 4E,F). These results showed that microglia-Exos have a neuroprotective effect and could protect neurons from apoptosis induced by SCI. To investigate whether microglia-Exos could promote axon regrowth *in vivo*, we performed fluorescence staining with MAP2 and 5-hydroxytryptamine (5-HT), separately. We found that microglia-Exos could increase the MAP2-positive area (Figures 4G,H) and the 5-HT–positive area (Supplementary Figure S3A,B) compared with that in the control group. Collectively, these data suggested that microglia-Exos could protect against neuron loss and facilitate axon regrowth by suppressing neuronal apoptosis.

### miR-151-3p Is Enriched in Microglia-Exos and Transferred to Neurons

**FIGURE 5:**
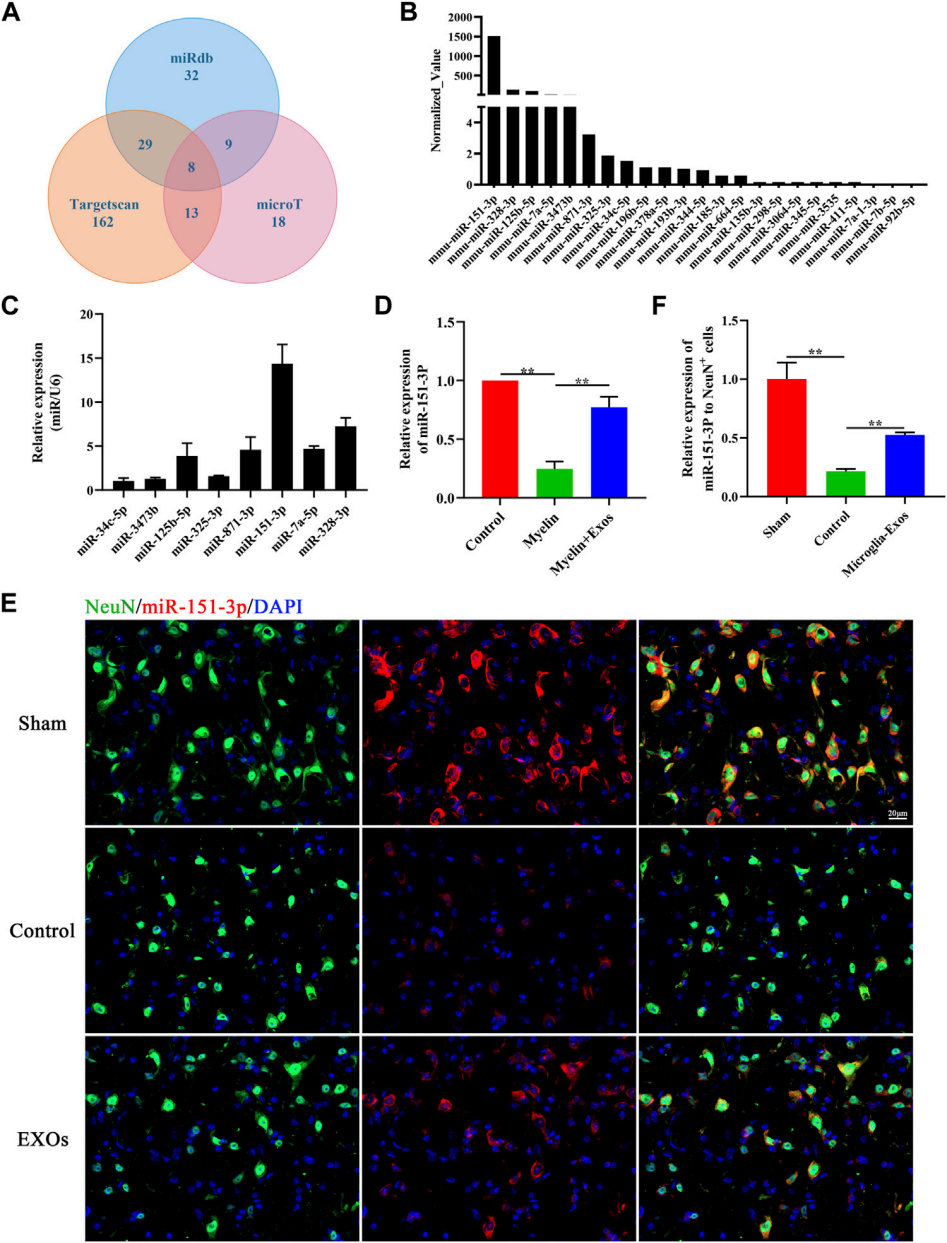
miR-151-3p is enriched in Microglia-Exos and transfer to neurons**. (A)** Predicted microRNAs targeted to p53 by TargetScan, micro T, and miRDB databases. **(B)** Top 23 highly expressed microRNAs of microglia-Exos that targeted to p53 are listed. **(C)** qRT-PCR analysis of the top eight expressed microRNAs in Microglia-Exos. N = 3 per group. **(D)** Relative expression of miR-151-3p in control, myelin debris, and myelin debris + Microglia-Exo–treated neurons by qRT-PCR. N = 3 per group. **(E)** *In situ* hybridization of miR-151-3p (red) and fluorescence staining of NeuN (green) of sham, control, and microglia-Exo–treated groups at the lesion site 3 days post-SCI. Scale bar: 20 μm. **(F)** Quantification of relative expression of miR-151-3p to NeuN^+^ cells in **(E)**. N = 3 per group. Data are presented as the mean ± SD. **p < 0.01.

To explore the key components of microglia-Exo cargo that mediate the neuroprotective effects of microglia-Exos on neurons, we comprehensively analyzed the miRDB, TargetScan, and microT databases. We found 169 potential microRNAs that could bind to p53 (Figure 5A). By comparing the microRNA expression profiles of microglia-Exos in public databases, the top 23 highly expressed microRNAs were selected and are listed in Figure 5B. We chose the top eight expressed microRNAs for our research of interest and validated them using qRT-PCR. Among them, the relative expression of miR-151-3p was highest in microglia-Exo cargo (Figure 5C). To further determine whether microglia-Exos could deliver miR-151-3p to neurons, we cultured neurons by adding microglia-Exos and performed qRT-PCR to measure the expression level of miR-151-3p in neurons. We found that the expression level of miR-151-3p in neurons was markedly decreased with added myelin debris and was increased under treatment with microglia-Exos (Figure 5D). Similar to the real-time PCR results, the FISH experiment for miR-151-3p detection in the *in vivo* SCI model showed the same trend. As shown in Figure 5E, miR-151-3p was mainly expressed in neurons, and it was downregulated in the SCI groups. Microglia-Exo administration significantly increased miR-151-3p in neurons compared with the control groups. Collectively, these results suggested that microglia-Exos could deliver miR-151-3p to neurons and increase the expression of miR-151-3p in neurons.

### Microglial Exosomal miR-124-3p Inhibits Neuronal Apoptosis and Promotes Axonal Growth by Suppressing the p53/p21/CDK1 Signaling Pathway

**FIGURE 6:**
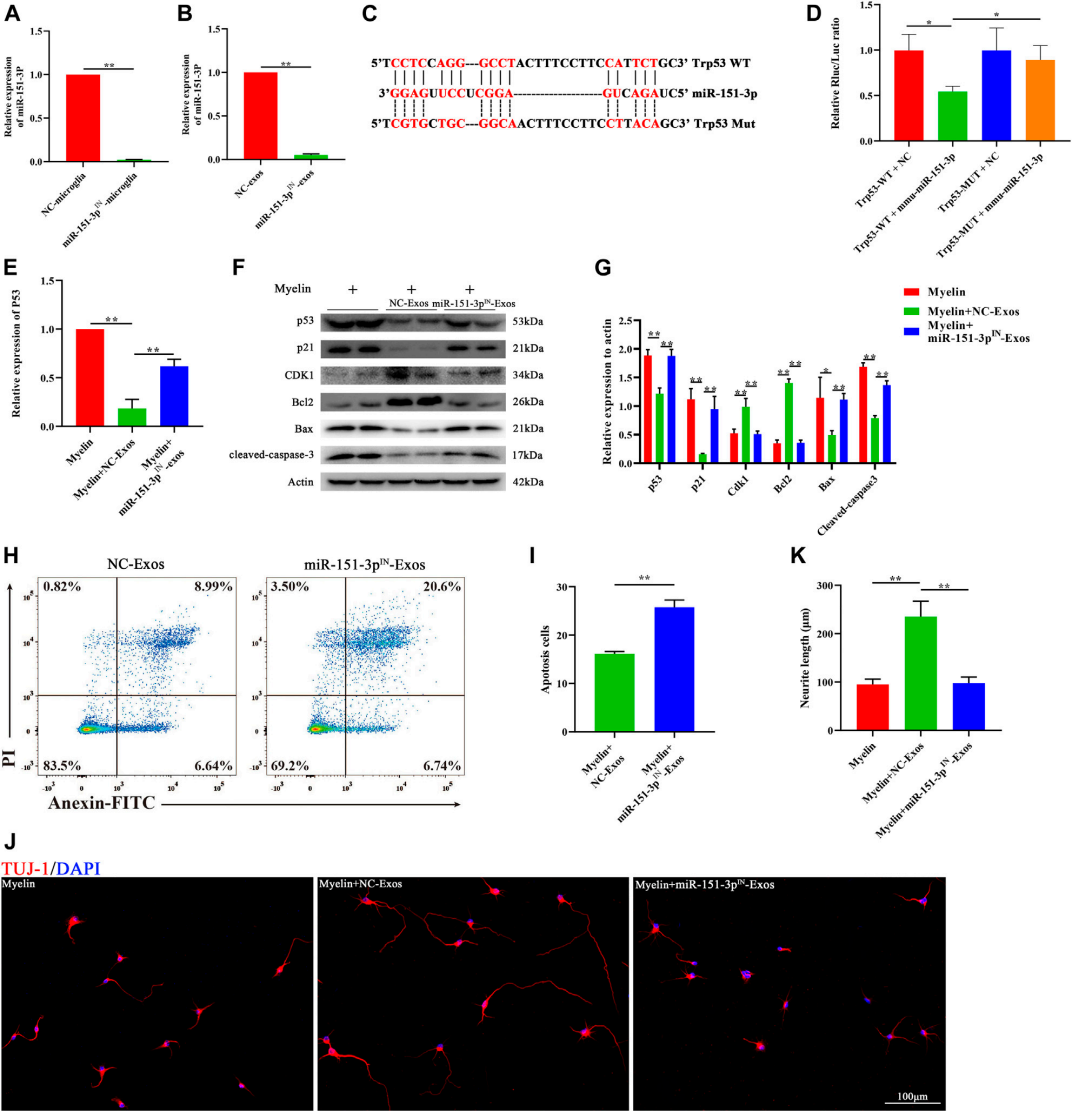
Microglia-Exos rescue neuron apoptosis and promote neurite growth *via* suppressing p53/p21/CDK1 signaling through miR-151-3p**. (A)** Relative expression of miR-151-3p of negative control and miR-151-3p inhibitor transfected microglia cells (NC-microglia and miR-151-3p^IN^-microglia) by qRT-PCR. N = 3 per group. **(B)** Relative expression of miR-151-3p of exosomes derived from negative control and miR-151-3p inhibitor transfected microglia cells (NC-Exos and miR-151-3p^IN^-Exos) by qRT-PCR. N = 3 per group. **(C)** Complementary sequences between miR-151-3p and the 3′ UTR of Trp53. **(D)** Relative luciferase activities of the p53-wt + negative control group, p53-wt + miR-151-3p group, p53-mut + negative control group, and p53-mut + miR-151-3p group. N = 3 per group. **(E)** Relative expression of p53 of myelin debris, myelin debris + microglia-Exos, and myelin debris + miR-151-3p^IN^-Exo–treated groups by qRT-PCR. N = 3 per group. **(F)** Western blot analysis of p53, p21, CDK1, apoptosis-related protein Bax, and cleaved-caspase-3 and anti-apoptosis protein Bcl2 in myelin debris, myelin debris + Microglia-Exos, and myelin debris + miR-151-3p^IN^-Microglia-Exo–treated groups. **(G)** Quantification of proteins’ relative expression in **(F)**. N = 3 per group. **(H)** Flow cytometry of Annexin/PI staining in myelin debris, myelin debris + Microglia-Exos, and myelin debris + miR-151-3p^IN^-Microglia-Exo–treated groups. **(I)** Quantification of the apoptosis rate of neurons in **(D)**. N = 3 per group. **(J)** Representative fluorescent images of neuron staining with Tuj-1 (red) of myelin debris, myelin debris + Microglia-Exos, and myelin debris + miR-151-3p^IN^-Microglia-Exo–treated groups. Scale bar: 100 μm. **(K)** Quantification of neurite length in **(J)**. N = 5 per group. Data are presented as the mean ± SD. *p < 0.05, **p < 0.01.

To investigate the function of exosomal miR-151-3p in mediating the neuroprotective effect of microglia-Exos on neuronal and axonal growth, we constructed a miR-151-3p inhibitor (miR-151-3p^IN^) and transfected it into microglia. Microglia cells transfected with the miR-151-3p inhibitor (miR-151-3p^IN^ microglia) or the miR-151-3p negative control (miR-151-3p^NC^ microglia) showed no difference in morphology (Supplementary Figure S1A). TEM, nanotracking analysis, and Western blot were used to identify the exosomes isolated from miR-151-3p^IN^ microglia and miR-151-3p^NC^ microglia, and we found no difference in exosome diameters and surface markers between the two groups (Supplementary Figure S1B,D). We then extracted RNA from microglial cells and microglial exosomes for qRT–PCR to verify the inhibitory effect of the miR-151-3p inhibitor. As shown in Figure 6A and Figure 6B, the expression of miR-151-3p in microglia and microglia-Exos was obviously decreased 3 days after transfection with the miR-151-3p inhibitor. The 3′UTR of p53 shared a complementary sequence of miR-151-3p (Figure 6C). The dual luciferase reporter assay was used to examine the binding relationship between miR-151-3p and p53. As presented in Figure 6D, miR-151-3p reduced luciferase activity in cells carrying wild-type p53 plasmids when compared with the miR-151-3p negative control (NC). Furthermore, no change in luciferase activity was seen in cells carrying mutant p53 plasmids. These results indicated that miR-151-3p could directly bind to p53.

To investigate whether exosomal miR-151-3p was involved in the downregulation of the p53/p21 signaling pathway and apoptosis-related proteins in neurons, we added microglia-Exos or miR-151-3p inhibitor-transfected microglia-derived exosomes (miR-151-3p^IN−^Exos). As shown in Figure 6E, qRT–PCR data indicated that miR-151-3p^IN−^Exos could upregulate p53 gene expression in neurons, which was downregulated in the microglia-Exos treatment groups. Similar to the qRT–PCR results, Western blot analysis showed that miR-151-3p^IN−^Exos obviously abolished the effect of microglia-Exos at the protein level of p53. (Figures 6F,G). In addition to p53, the miR-151-3p^IN^-Exos promoted the protein expression of p21 and p53, upregulated the expression of apoptosis-related proteins, such as Bax and cleaved caspase-3, and downregulated the expression of the antiapoptotic protein Bcl2 (Figures 6F,G). Flow cytometry for neuronal apoptosis demonstrated that miR-151-3p^IN−^Exos abolished the effect of microglia-Exos on the inhibition of neuronal apoptosis (Figures 6H,I). Immunofluorescence staining indicated that miR-151-3p^IN−^Exos abolished the ability of microglia-Exos to promote axon growth (Figures 6J,K). Taken together, these results demonstrated that microglial exosomal miR-124-3p inhibited neuronal apoptosis and promoted axonal growth by suppressing p53 by directly targeting its 3′-UTR and thereby modulating the p53/p21/CDK1 signaling pathway.

### Exosomal miR-151-3p Mediates the Neuroprotective Effects of Microglia-Exos on SCI

**FIGURE 7:**
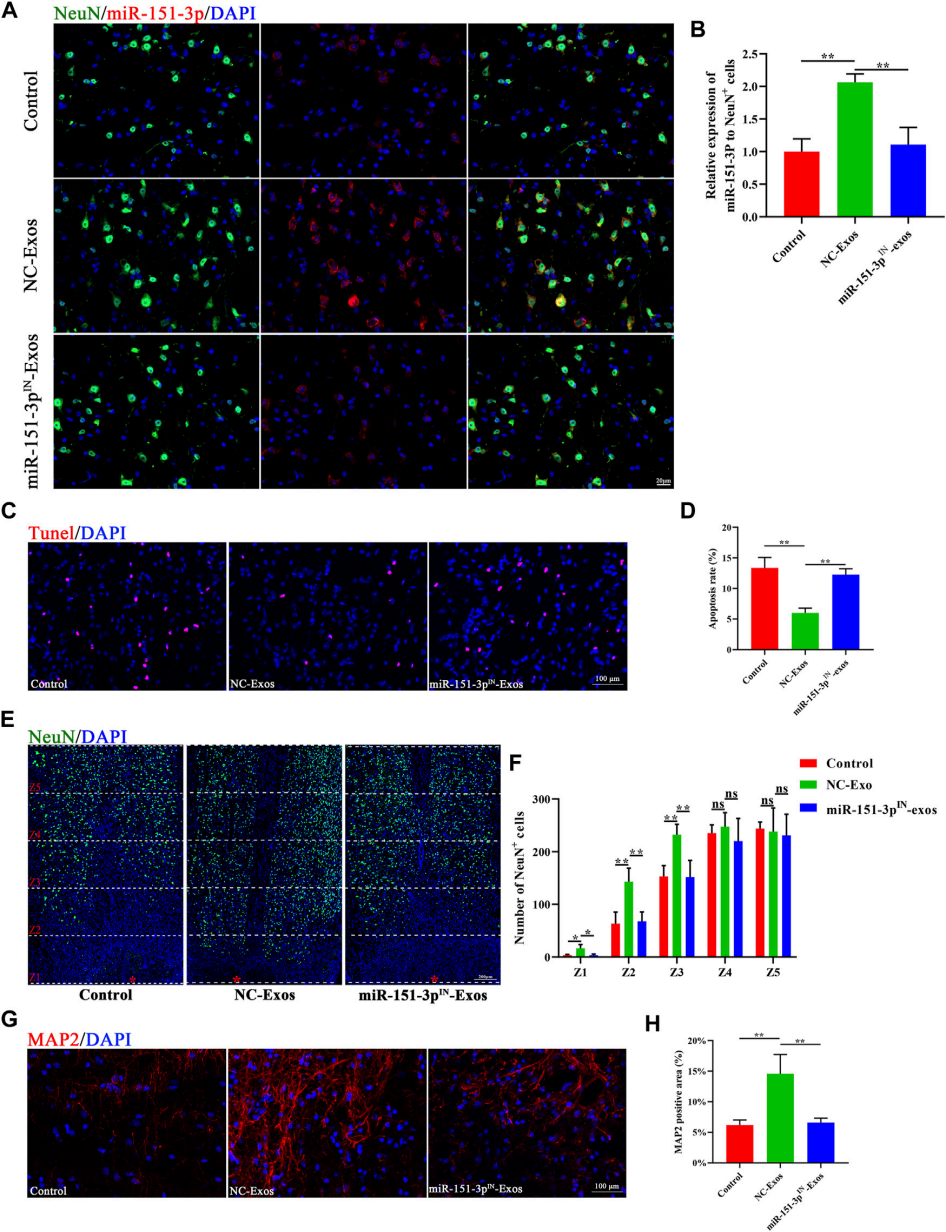
Exosomal miR-151-3p mediate the neuro-protective effects of microglia-Exos on SCI. **(A)** *In situ* hybridization of miR-151-3p (red) and fluorescence staining of NeuN (green) of control, Microglia-Exos, and miR-151-3p^IN^- Exo–treated groups at the lesion site 3 days post-SCI. Scale bar: 20 μm. **(B)** Quantification of relative expression of miR-151-3p to NeuN + cells in (E). N = 3 per group. **(C)** Representative images of TUNEL staining of control, microglia-exosomes, and miR-151-3p^IN^-Exo–treated groups. Scale bar: 100 μm. **(D)** Quantification of the apoptosis rate in **(C)**. N = 4 per group. **(E)** Representative staining images of NeuN (green) of control, microglia-Exos, and miR-151-3p^IN^-Exo–treated groups at different zones. The red star indicates the center of the injury site. Scale bar: 200 μm. **(F)** Quantification of number of NeuN-positive cells at different zones in **(E)**. N = 4 per group. **(G)** Representative staining images of MAP2 (red) of control, microglia-Exos, and miR-151-3p^IN^-Exo–treated groups. Scale bar: 100 μm. **(H)** Quantification of the MAP2-positive area in **(G)**. N = 4 per group. Data are presented as the mean ± SD. *p < 0.05, **p < 0.01, ns = not significant.

**FIGURE 8:**
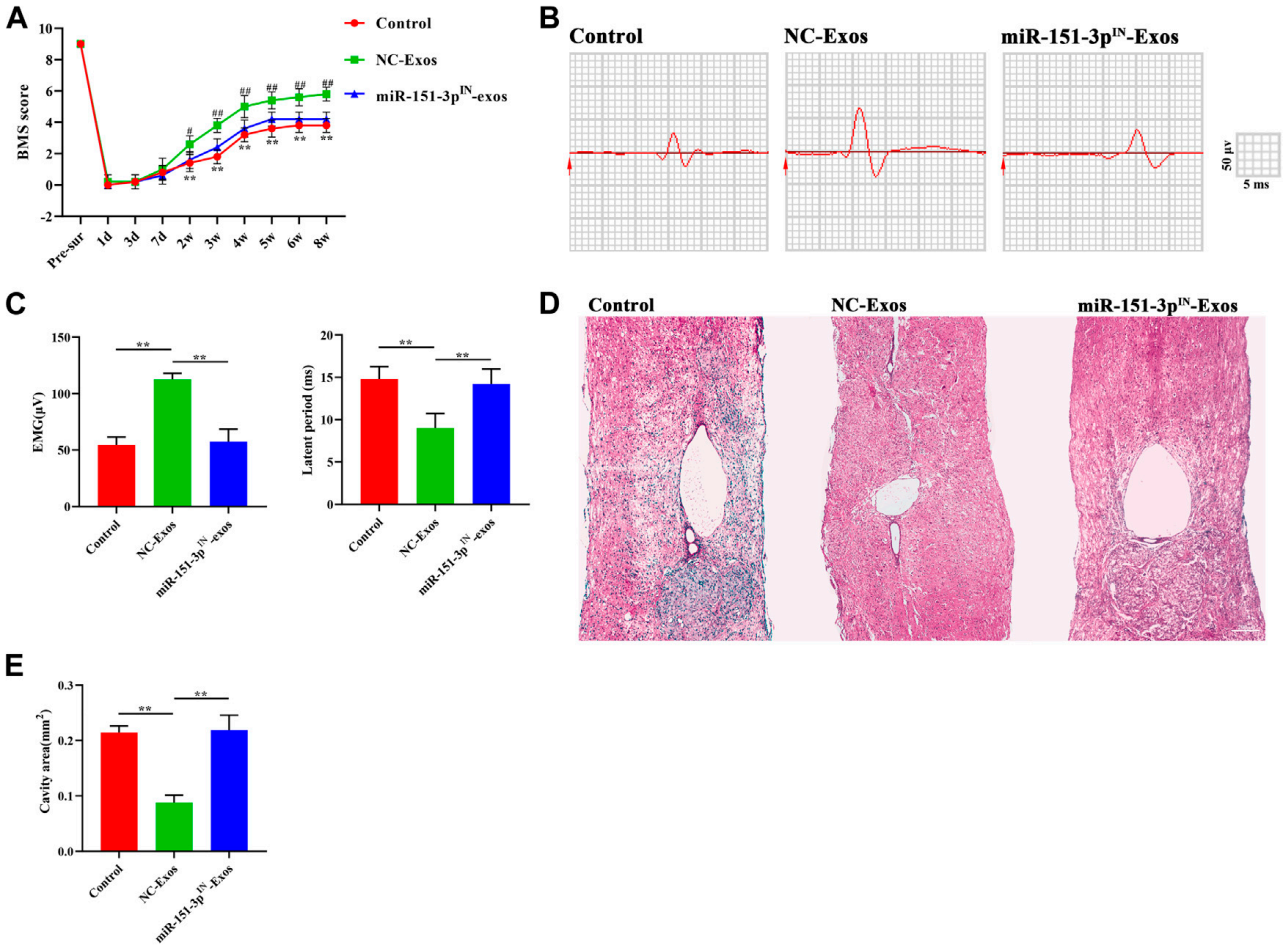
Microglia-Exos promote neurological function healing after SCI *via* transfer of miR-151-3p**. (A)** BMS scores in control, Microglia-Exos, and miR-151-3p^IN^- Microglia-Exo–treated groups at different time points. N = 5 per group. **(B)** Neuroelectrophysiology analysis of control, microglia-Exos, and miR-151-3p^IN^-Exo–treated groups at 8 weeks after injury. **(C)** Quantification of motor evoked potential (MEP) and latent period in **(B)**. N = 5 per group. **(D)** Representative images of HE staining of control, microglia-Exos, and miR-151-3p^IN^-Exo–treated groups. Scale bar: 200 μm. **(E)** Quantification of the cavity area of **(D)**. N = 5 per group. Data are presented as the mean ± SD. *p < 0.05, **p < 0.01.

To explore the protective effect of exosomal miR-151-3p on the neuroprotection of microglia-Exos in the injured spinal cord, we injected microglia-Exos or miR-151-3p^IN-^microglia-Exos *via* the tail vein into SCI mice. The expression of miR-151-3p in neurons was significantly lower in the miR-151-3p^IN^-Exo–treated group (Figures 7A,B) and was associated with more apoptotic cells and neuronal loss than the microglia-Exo–treated group (Figures 7C–F). For axon visualization, the mice treated with miR-151-3p^IN^-Exos showed a smaller MAP2-positive area (Figures 7G,H) and 5-HT–positive area (Supplementary Figure S3C,D) than the microglia-Exos (NC-Exos)-treated group. To determine whether miR-151-3p was involved in microglia-Exo–mediated neurological function healing after SCI, we conducted BMS score and neuro-electrophysiology analyses. After SCI, the BMS score in each group increased gradually, indicating that mice had a certain self-recovery ability. Compared with the microglia-Exo–treated (NC-Exos) group, the recovery of the miR-151-3p^IN^-Exo–treated group was relatively worse from the second week until the endpoint of observation (Figure 8A). The results of neuro-electrophysiology analysis showed similar results, and miR-151-3p^IN^-Exos abolished the beneficial effect of microglia-Exos on promoting neurological functional healing after SCI and demonstrated a decreased MEP and increased latent period when miR-151-3p was downregulated in microglia-derived exosomes (Figures 8B,C). Histological analysis of HE-stained spinal cord sections showed a larger cavity area in miR-151-3p^IN^-Exo–treated mice than in microglia-Exo–treated mice (Figures 8D,E). Therefore, these results indicated that exosomal miR-124-3p mediates microglia-Exos to promote neurological functional healing effects after SCI.

## Discussion

Many approaches have been conducted to promote functional neurological healing after SCI, but optimal and effective treatments are still lacking and are being developed. Our present study demonstrated that exosomes derived from microglial cells could be taken up by neurons and further inhibited neuronal apoptosis, promoted axonal growth, and finally facilitated functional neurological recovery after SCI. This neuroprotection process is mediated by microglia-exosomal miR-151-3p and its target gene p53 expression in neurons. Microglia-derived exosomal microRNA-151-3p enhances neurological functional healing after spinal cord injury by promoting axon growth and attenuating neuronal apoptosis through regulating the p53/p21/CDK1 signaling pathway. Microglia-Exo-based cell-free therapy provides a novel therapeutic strategy and suggests a better option for SCI repair.

Exosomes can be secreted by multiple cells, such as immune cells, and exosome treatment shows similar therapeutic effects to direct transplantation of parental cells (Dutta et al., 2021). Exosomes also have nonimmune rejection, nontumorigenicity, high stability, and cell targeting. In addition, exosomes may penetrate the blood–brain barrier (BBB) and retain their biological activity (Elliott and He 2021). These advantages make exosomes a cell-free therapy that is an attractive approach for SCI treatment. As the main immune cells in the central nervous system (CNS), the role of microglia in spinal cord injury (SCI) remains unclear. Together with blood-borne monocyte-derived macrophages, microglia constitute the innate immunity responsible for providing a source of anti-inflammatory factors to promote tissue repair in the CNS (Beck et al., 2010). In the event of traumatic injury in the CNS, microglia are the first cell type to rapidly respond and extend cytoplasmic processes toward the lesion site to form a protective barrier that limits neural tissue damage (Davalos et al., 2005; Zhou et al., 2020). This barrier contains inflammatory cytokines, facilitates debris clearing, and blocks the spread of damage in the brain (Popovich and Jones 2003; Hines et al., 2009). Following traumatic insults, microglia undergoing highly diverse changes could be polarized into either the “M1 classically activated” phenotype or the “M2 alternatively activated” phenotype and play a critical role in mediating functional healing (Kigerl et al., 2009; Hakim et al., 2021). In a spinal cord injury model, Fu et al. found that microglia depletion enhanced immune cell infiltration and impaired locomotor recovery after SCI (Fu et al., 2020). In a mouse model of contusive SCI, microglia have also been identified as a key cellular component for scar formation that could protect neural tissue after SCI (Bellver-Landete et al., 2019). This evidence reinforces the idea that the overall effect of microglia in the CNS after injury is neuroprotective. The beneficial effect of microglia on functional neurological protection suggests that it will therefore be highly of interest to study the biological properties of exosomes derived from microglia. Our previous study found that microglia-Exos exerted an antioxidant effect and could promote endothelial cell survival and thus positively modulate vascular regeneration and promote neurological functional recovery post-SCI (Peng et al., 2021).

Our current study reported an interaction between microglia and neurons and demonstrated that microglia-Exos could be taken up by neurons both *in vivo* and *in vitro*. Microglia-Exos can affect neuronal apoptosis and promote axonal growth. Our neurological function assessment also revealed that Micro-Exos promote neurological function recovery after SCI. These results indicated that in addition to vascular endothelial cells. We unveiled an additional beneficial effect of Microglia-Exos on neurological functional recovery after SCI that is mainly attributable to their protective effects on neuronal cells. Thus far, the protective effects of microglia-Exos on neurons are fully understood; however, the underlying signaling mechanisms by which microglia-Exos contribute to inhibiting neuronal apoptosis and promoting axonal growth are unclear and need further exploration.

Neuronal apoptosis has been documented in the rodent central nervous system (CNS) following spinal cord injury. Trauma-induced spinal neuron apoptosis is p53- and Bax-dependent (Kotipatruni et al., 2011). p53-mediated neuronal apoptosis occurs through various molecular mechanisms in CNS injury (Hong et al., 2010). The cyclin-dependent kinase (CDK) inhibitor p21 is one of the factors that can promote cell cycle arrest in response to various stimuli. p21 can be induced by both p53-dependent and p53-independent mechanisms (Karimian et al., 2016). p53-p21 pathway activation in response to DNA damage inhibits cyclin-dependent kinase (CDK) activities, resulting in cell apoptosis (Karimian et al., 2016). Growing evidence has reported that axon growth and neuronal apoptosis are crucial therapeutic targets for SCI treatments. In a C9orf72 mouse mode which usually causes amyotrophic lateral sclerosis (ALS) and frontotemporal dementia (FTD), ablating P53 could rescue axon degeneration and promoted neuron survival (Maor-Nof et al., 2021). Our study found that microglia-Exos reduced neuronal apoptosis and suppressed the p53/p21/CDK1 signaling pathway. Western blotting data showed that the downstream pro-apoptotic proteins Bax and cleaved caspase-3 were downregulated, while the antiapoptotic protein Bcl2 was upregulated after the administration of microglia-Exos.

Exosomes exert their effect by transferring exosomal functional cargo (miRNAs, RNAs, DNAs, and proteins) to recipient cells (Feng et al., 2021). Small miRNAs representing a significant proportion of the exosome cargo have attracted substantial attention. miRNAs are a class of single-stranded ncRNAs and can promote mRNA degradation by directly binding to the 3′-UTR (untranslated region) of their target mRNAs. In this study, we found 169 potential microRNAs that could bind to Tp53 and compared the microRNA expression profiles of microglia-Exos in public databases, among which 23 microRNAs expressed in microglia cells according to the online dataset and miR-151-3p was highly expressed in microglia-Exo cargo content. As initially observed in an animal model of retinopathy of prematurity, microRNA-151-3p was first reported to be expressed in exosomes derived from microglia (Xu et al., 2019). Multiple miRNAs in exosomes for SCI treatment has been explored. The miR-21–rich exosomes could enhance neuronal viability by downregulating the PTEN/PDCD4 expression after SCI (Kang et al., 2019). The miR-216a-5p–encapsulated exosomes derived from MSC under hypoxia could promote the functional recovery after SCI by activating the microglial polarization (Liu et al., 2020). However, the function of microRNA-151-3p in microglia was not explored. In this study, we confirmed that silencing miR-151-3p in microglia-Exos attenuated the inhibitory effect of microglia-Exos on neuronal apoptosis. Multiple target genes for miR-151-3p include activating death-associated protein kinase 1 (DAPK-1) Stat3 and p53. Our experiments showed miR-151-3p as a class of single-stranded ncRNAs that can promote Tp53 degradation by directly binding to the 3′-UTR (untranslated region). These results indicate that exosomal miR-151-3p is the key miRNA by which microglia-Exos exert their neuroprotective effect on SCI. Although the inhabitation of miR-151-3p in microglia-Exos mostly abolished the effect of microglia-derived exosome in SCI treatment, considering our previous studies showed that microglia-Exos can also improve spinal cord functional recovery *via* inhibiting oxidative stress in endothelia cells and miR-151-3p^IN^–transfected microglia may bring other unexpected toxic factors in the exosomes, the multiple roles of microglia-Exos still need further exploration (Peng et al., 2021).

After an injury, spinal cord neurons exhibit excess apoptosis and degeneration and display impaired axonal growth (Han et al., 2020). In our study, we found that in addition to inhibiting neuronal apoptosis by microglia-Exos, exosome treatment could also promote axonal growth, and microglial exosomal miR-151-3p mediated this process. However, whether inhibiting neuronal apoptosis can activate axonal growth to prevent tissue damage has not been reported. We hypothesize that the inhibition of neuronal apoptosis may create a regenerative microenvironment for axon growth. However, the internal relationship and undying mechanism between neuronal apoptosis and axonal growth should be further explored.

In summary, our study revealed that microglia-Exos promote neurological functional recovery after SCI by shuttling exosomal miR-151-3p to neurons following SCI in mice. High expression levels of exosomal miR-151-3p in microglia-Exos exhibit neuroprotective effects by suppressing neuronal apoptosis and promoting axonal growth *via* the p53/p21/CDK1 signaling pathway. Cell-free and nanosized microglia-Exos show promising potential as an effective therapeutic intervention for delivering functional miRNA into the injured spinal cord and promoting functional recovery after SCI.

## Data Availability

The original contributions presented in the study are included in the article/Supplementary Material; further inquiries can be directed to the corresponding authors.

## References

[B1] Ahuja C. S. Nori S. Tetreault L. Wilson J. Kwon B. Harrop J. (2017a). Traumatic Spinal Cord Injury-Repair and Regeneration. Neurosurgery 80 (3S), S9. 10.1093/neuros/nyw080 28350947

[B2] Ahuja C. S. Wilson J. R. Nori S. Kotter M. R. N. Druschel C. Curt A. (2017b). Traumatic Spinal Cord Injury. Nat. Rev. Dis. Primers 3, 17018. 10.1038/nrdp.2017.18 28447605

[B3] Aoki S. Kong D. Suna H. Sowa Y. Sakai T. Setiawan A. (2006). Aaptamine, a Spongean Alkaloid, Activates P21 Promoter in a P53-independent Manner. Biochem. Biophysical Res. Commun. 342 (1), 101–106. 10.1016/j.bbrc.2006.01.119 16480688

[B4] Basso D. M. Fisher L. C. Anderson A. J. Jakeman L. B. McTigue D. M. Popovich P. G. (2006). Basso Mouse Scale for Locomotion Detects Differences in Recovery after Spinal Cord Injury in Five Common Mouse Strains. J. neurotrauma 23 (5), 635–659. 10.1089/neu.2006.23.635 16689667

[B5] Beck K. D. Nguyen H. X. Galvan M. D. Salazar D. L. Woodruff T. M. Anderson A. J. (2010). Quantitative Analysis of Cellular Inflammation after Traumatic Spinal Cord Injury: Evidence for a Multiphasic Inflammatory Response in the Acute to Chronic Environment. Brain 133 (Pt 2), 433–447. 10.1093/brain/awp322 20085927 PMC2858013

[B6] Bellver-Landete V. Bretheau F. Mailhot B. Vallières N. Lessard M. Janelle M.-E. (2019). Microglia Are an Essential Component of the Neuroprotective Scar that Forms after Spinal Cord Injury. Nat. Commun. 10 (1), 518. 10.1038/s41467-019-08446-0 30705270 PMC6355913

[B7] Davalos D. Grutzendler J. Yang G. Kim J. V. Zuo Y. Jung S. (2005). ATP Mediates Rapid Microglial Response to Local Brain Injury *In Vivo* . Nat. Neurosci. 8 (6), 752–758. 10.1038/nn1472 15895084

[B8] Du S. Xiong S. Du X. Yuan T. F. Peng B. Rao Y. (2021). Primary Microglia Isolation from Postnatal Mouse Brains. J. Vis. Exp. (168). 10.3791/62237 33720125

[B9] Dutta D. Khan N. Wu J. Jay S. M. (2021). Extracellular Vesicles as an Emerging Frontier in Spinal Cord Injury Pathobiology and Therapy. Trends Neurosciences 44 (6), 492–506. 10.1016/j.tins.2021.01.003 PMC8159852 33581883

[B10] Elliott R. O. He M. (2021). Unlocking the Power of Exosomes for Crossing Biological Barriers in Drug Delivery. Pharmaceutics 13 (1), 122. 10.3390/pharmaceutics13010122 33477972 PMC7835896

[B11] Feng J. Zhang Y. Zhu Z. Gu C. Waqas A. Chen L. (2021). Emerging Exosomes and Exosomal MiRNAs in Spinal Cord Injury. Front. Cel Dev. Biol. 9, 703989. 10.3389/fcell.2021.703989 PMC8299525 34307384

[B12] Fu H. Zhao Y. Hu D. Wang S. Yu T. Zhang L. (2020). Depletion of Microglia Exacerbates Injury and Impairs Function Recovery after Spinal Cord Injury in Mice. Cell Death Dis 11 (7), 528. 10.1038/s41419-020-2733-4 32661227 PMC7359318

[B13] GSchlag Hopf R. Redl H. (2001). Serial Recording of Sensory, Corticomotor, and Brainstem-Derived Motor Evoked Potentials in the Rat. Somatosensory Mot. Res. 18 (2), 106–116. 10.1080/135578501012006219 11534774

[B14] Guo M. Hao Y. Feng Y. Li H. Mao Y. Dong Q. (2021a). Microglial Exosomes in Neurodegenerative Disease. Front. Mol. Neurosci. 14, 630808. 10.3389/fnmol.2021.630808 34045943 PMC8148341

[B15] Guo Z. Li C. Cao Y. Qin T. Jiang L. Xu Y. (2021b). UTX/KDM6A Deletion Promotes the Recovery of Spinal Cord Injury by Epigenetically Triggering Intrinsic Neural regenerationMolecular Therapy. Mol. Ther. Methods Clin. Dev. 20, 337–349. 10.1016/j.omtm.2020.12.004 33553483 PMC7820127

[B16] Hakim R. Zachariadis V. Sankavaram S. R. Han J. Harris R. A. Brundin L. (2021). Spinal Cord Injury Induces Permanent Reprogramming of Microglia into a Disease-Associated State Which Contributes to Functional Recovery. J. Neurosci. 41 (40), 8441–8459. 10.1523/jneurosci.0860-21.2021 34417326 PMC8496189

[B17] Hamzah R. N. Alghazali K. M. Biris A. S. Griffin R. J. (2021). Exosome Traceability and Cell Source Dependence on Composition and Cell-Cell Cross Talk. Int. J. Mol. Sci. 22 (10), 5346. 10.3390/ijms22105346 34069542 PMC8161017

[B18] Han W. Li Y. Cheng J. Zhang J. Chen D. Fang M. (2020). Sitagliptin Improves Functional Recovery *via* GLP-1r-Induced Anti-apoptosis and Facilitation of Axonal Regeneration after Spinal Cord Injury. J. Cel Mol Med 24 (15), 8687–8702. 10.1111/jcmm.15501 PMC7412681 32573108

[B19] Hao D. Du J. Yan L. He B. Qi X. Yu S. (2021). Trends of Epidemiological Characteristics of Traumatic Spinal Cord Injury in China, 2009-2018European Spine Journal. Eur. Spine Jand Eur. Section Cervical Spine Res. Soc. 30 (10), 3115–3127. 10.1007/s00586-021-06957-3 34392419

[B20] Hines D. J. Hines R. M. Mulligan S. J. Macvicar B. A. (2009). Microglia Processes Block the Spread of Damage in the Brain and Require Functional Chloride Channels. Glia 57 (15), 1610–1618. 10.1002/glia.20874 19382211

[B21] Hong L.-Z. Zhao X.-Y. Zhang H.-L. (2010). p53-mediated Neuronal Cell Death in Ischemic Brain Injury. Neurosci. Bull. 26 (3), 232–240. 10.1007/s12264-010-1111-0 20502500 PMC5560294

[B22] Hou B.-R. Jiang C. Wang Z.-N. Ren H.-J. (2020). Exosome-mediated Crosstalk between Microglia and Neural Stem Cells in the Repair of Brain Injury. Neural Regen. Res. 15 (6), 1023–1024. 10.4103/1673-5374.270302 31823874 PMC7034280

[B23] Kang J. Li Z. Zhi Z. Wang S. Xu G. (2019). MiR-21 Derived from the Exosomes of MSCs Regulates the Death and Differentiation of Neurons in Patients with Spinal Cord Injury. Gene Ther. 26 (12), 491–503. 10.1038/s41434-019-0101-8 31570818

[B24] Karimian A. Ahmadi Y. Yousefi B. (2016). Multiple Functions of P21 in Cell Cycle, Apoptosis and Transcriptional Regulation after DNA Damage. DNA repair 42, 63–71. 10.1016/j.dnarep.2016.04.008 27156098

[B25] Khorasanizadeh M. Yousefifard M. Eskian M. Lu Y. Chalangari M. Harrop J. S. (2019). Neurological Recovery Following Traumatic Spinal Cord Injury: a Systematic Review and Meta-Analysis. J. Neurosurg. Spine, 1–17. 10.3171/2018.10.spine18802 30771786

[B26] Kigerl K. A. Gensel J. C. Ankeny D. P. Alexander J. K. Donnelly D. J. Popovich P. G. (2009). Identification of Two Distinct Macrophage Subsets with Divergent Effects Causing Either Neurotoxicity or Regeneration in the Injured Mouse Spinal Cord. J. Neurosci. 29 (43), 13435–13444. 10.1523/jneurosci.3257-09.2009 19864556 PMC2788152

[B27] Kotipatruni R. R. Dasari V. R. Veeravalli K. K. Dinh D. H. Fassett D. Rao J. S. (2011). p53- and Bax-Mediated Apoptosis in Injured Rat Spinal Cord. Neurochem. Res. 36 (11), 2063–2074. 10.1007/s11064-011-0530-2 21748659

[B28] Li M. Rong Z.-J. Cao Y. Jiang L.-Y. Zhong D. Li C.-J. (2021). Utx Regulates the NF-Κb Signaling Pathway of Natural Stem Cells to Modulate Macrophage Migration during Spinal Cord Injury. J. neurotrauma 38 (3), 353–364. 10.1089/neu.2020.7075 32977735

[B29] Li Y. He X. Kawaguchi R. Zhang Y. Wang Q. Monavarfeshani A. (2020). Microglia-organized Scar-free Spinal Cord Repair in Neonatal Mice. Nature 587 (7835), 613–618. 10.1038/s41586-020-2795-6 33029008 PMC7704837

[B30] Liu W. Rong Y. Wang J. Zhou Z. Ge X. Ji C. (2020). Exosome-shuttled miR-216a-5p from Hypoxic Preconditioned Mesenchymal Stem Cells Repair Traumatic Spinal Cord Injury by Shifting Microglial M1/M2 Polarization. J. Neuroinflammation 17 (1), 47. 10.1186/s12974-020-1726-7 32019561 PMC7001326

[B31] Maor-Nof M. Shipony Z. Lopez-Gonzalez R. Nakayama L. Zhang Y.-J. Couthouis J. (2021). p53 Is a central Regulator Driving Neurodegeneration Caused by C9orf72 Poly(PR). Cell 184 (3), 689–708. e620. 10.1016/j.cell.2020.12.025 33482083 PMC7886018

[B32] Peng W. Wan L. Luo Z. Xie Y. Liu Y. Huang T. (2021). Microglia-Derived Exosomes Improve Spinal Cord Functional Recovery after Injury via Inhibiting Oxidative Stress and Promoting the Survival and Function of Endothelia Cells. Oxid Med. Cel Longev 2021, 1695087. 10.1155/2021/1695087 PMC8413072 34484559

[B33] Popovich P. G. Jones T. B. (2003). Manipulating Neuroinflammatory Reactions in the Injured Spinal Cord: Back to Basics. Trends Pharmacological Sciences 24 (1), 13–17. 10.1016/s0165-6147(02)00006-8 12498725

[B34] Rolfe A. J. Bosco D. B. Broussard E. N. Ren Y. (2017). *In Vitro* Phagocytosis of Myelin Debris by Bone Marrow-Derived Macrophages. J. Vis. Exp. (130), 56322. 10.3791/56322 PMC5908409 29364206

[B35] Song Y. Li Z. He T. Qu M. Jiang L. Li W. (2019). M2 Microglia-Derived Exosomes Protect the Mouse Brain from Ischemia-Reperfusion Injury via Exosomal miR-124. Theranostics 9 (10), 2910–2923. 10.7150/thno.30879 31244932 PMC6568171

[B36] Sosa R. A. Murphey C. Robinson R. R. Forsthuber T. G. (2015). IFN-γ Ameliorates Autoimmune Encephalomyelitis by Limiting Myelin Lipid Peroxidation. Proc. Natl. Acad. Sci. USA 112 (36), E5038–E5047. 10.1073/pnas.1505955112 26305941 PMC4568689

[B37] Tran A. P. Warren P. M. Silver J. (2021). New Insights into Glial Scar Formation after Spinal Cord Injury. Cel Tissue Res. 10.1007/s00441-021-03477-w PMC8975767 34076775

[B38] Tran A. P. Warren P. M. Silver J. (2018). The Biology of Regeneration Failure and Success after Spinal Cord Injury. Physiol. Rev. 98 (2), 881–917. 10.1152/physrev.00017.2017 29513146 PMC5966716

[B39] Wu Y. Q. Xiong J. He Z. L. Yuan Y. Wang B. N. Xu J. Y. (2021). Metformin Promotes Microglial Cells to Facilitate Myelin Debris Clearance and Accelerate Nerve Repairment after Spinal Cord Injury. Acta Pharmacol. Sin. 10.1038/s41401-021-00759-5 PMC9160053 34480113

[B40] Xu W. Wu Y. Hu Z. Sun L. Dou G. Zhang Z. (2019). Exosomes from Microglia Attenuate Photoreceptor Injury and Neovascularization in an Animal Model of Retinopathy of Prematurity. Mol. Ther. - Nucleic Acids 16, 778–790. 10.1016/j.omtn.2019.04.029 31163320 PMC6545376

[B41] Zhang Y. Pan H.-Y. Hu X.-M. Cao X.-L. Wang J. Min Z.-L. (2016). The Role of Myocardin-Related Transcription Factor-A in Aβ 25-35 Induced Neuron Apoptosis and Synapse Injury. Brain Res. 1648 (Pt A), 27–34. 10.1016/j.brainres.2016.07.003 27387387

[B42] Zhou W. Silva M. Feng C. Zhao S. Liu L. Li S. (2021). Exosomes Derived from Human Placental Mesenchymal Stem Cells Enhanced the Recovery of Spinal Cord Injury by Activating Endogenous Neurogenesis. Stem Cel Res Ther 12 (1), 174. 10.1186/s13287-021-02248-2 PMC7953814 33712072

[B43] Zhou X. Wahane S. Friedl M.-S. Kluge M. Friedel C. C. Avrampou K. (2020). Microglia and Macrophages Promote Corralling, Wound Compaction and Recovery after Spinal Cord Injury via Plexin-B2. Nat. Neurosci. 23 (3), 337–350. 10.1038/s41593-020-0597-7 32112058 PMC7412870

