# Supplementary material for: Microglia-Derived Exosomal microRNA-151-3p Enhances Functional Healing After Spinal Cord Injury by Attenuating Neuronal Apoptosis via Regulating the p53/p21/CDK1 Signaling Pathway
Source: Front Cell Dev Biol. 2022 Jan 20;9:783017. doi: 10.3389/fcell.2021.783017 (PMC8811263; doi:10.3389/fcell.2021.783017)
Supplement: Supplementary file 1 [file DataSheet1.docx]

Table S1 Primers for qRT-PCR.

| Primers | Forward (5’-3’) | |
| --- | --- | --- |
| mmu-miR-34c-5p | CCAGGCAGTGTAGTTAGCTGATTGC | |
| mmu-miR-3473b | GGGCTGGAGAGATGGCTCA | |
| mmu-miR-125b-5p | TCTCCCTGAGACCCTAACTTGTGA | |
| mmu-miR-325-3p | TCGCCTTTATTGAGCACCTCCTATCAA | |
| mmu-miR-871-3p | CGTGACTGGCACCATTCTGGATAAT | |
| mmu-miR-151-3p | GCTAGACTGAGGCTCCTTGAGG | |
| mmu-miR-7a-5p | CGCCTGGAAGACTAGTGATTTTGTTGT | |
| mmu-miR-328-3p | CTGGCCCTCTCTGCCCTT | |
| Primers | Forward (5’-3’) | Reverse (5’-3’) |
| P53 | GTCACAGCACATGACGGAGG | TCTTCCAGATGCTCGGGATAC |
| GAPDH | GGTTGTCTCCTGCGACTTCA | TGGTCCAGGGTTTCTTACTCC |

Table S2 Primary antibodies

| Antibodies | Application and Dilution | Catalog numbers and species |
| --- | --- | --- |
| CD9 | WB: 1:1000 | Abcam (ab223052; Rabbit) |
| CD63 | WB: 1:1000 | Abcam (ab217345; Rabbit) |
| Tsg101 | WB: 1:1000 | Abcam (ab125011; Rabbit) |
| p53 | WB: 1:1000 | Wanleibio (WL01919; Rabbit) |
| p21 | WB: 1:1000 | Abcam (ab109199; Rabbit) |
| CDK1 | WB: 1:1000 | Proteintech (19532-1-AP; Rabbit) |
| Bcl-2 | WB: 1:1000 | Abcam (ab182858; Rabbit) |
| Bax | WB: 1:2000 | Abcam (ab32503; Rabbit) |
| IBA-1 | IF: 1:1000  F: 1:200 | Wako (019-19741; Rabbit)  Abcam (ab178846; Rabbit) |
| F4/80 | IF: 1:200 | Santa Cruz (sc-377009; Mouse) |
| Cleaved-caspase-3 | WB: 1:1000 | Abcam (ab214430; Rabbit) |
| Actin | WB: 1:2000 | Cell Signaling Technology (8457; Rabbit) |
| Tuj-1 | IF: 1:400 | Abcam (ab78078; Mouse) |
| MAP2 | IF: 1:500 | Abcam (ab32454; Rabbit) |
| 5-HT | IF: 1:5000 | ImmunoStar (20079; Goat) |
| NeuN | IF: 1:400 | Abcam (ab104224; Mouse) |
| Percp-cy5.5-CD11b | F: 1:200 | eBioscience™ (45-0112-82; Rat) |
| FITC-F4/80 | F: 1:200 | Biolegend (123107; Rat) |
| FITC-CD31 | F: 1:200 | Biolegend (102405; Rat) |
| PE-GLAST | F: 1:200 | Miltenyi Biotec (130-118-483; Mouse) |
| APC-O4 | F: 1:200 | Miltenyi Biotec (130-117-507,Mouse) |

WB: western blot; IF: immunofluorescence; F: flow cytometry


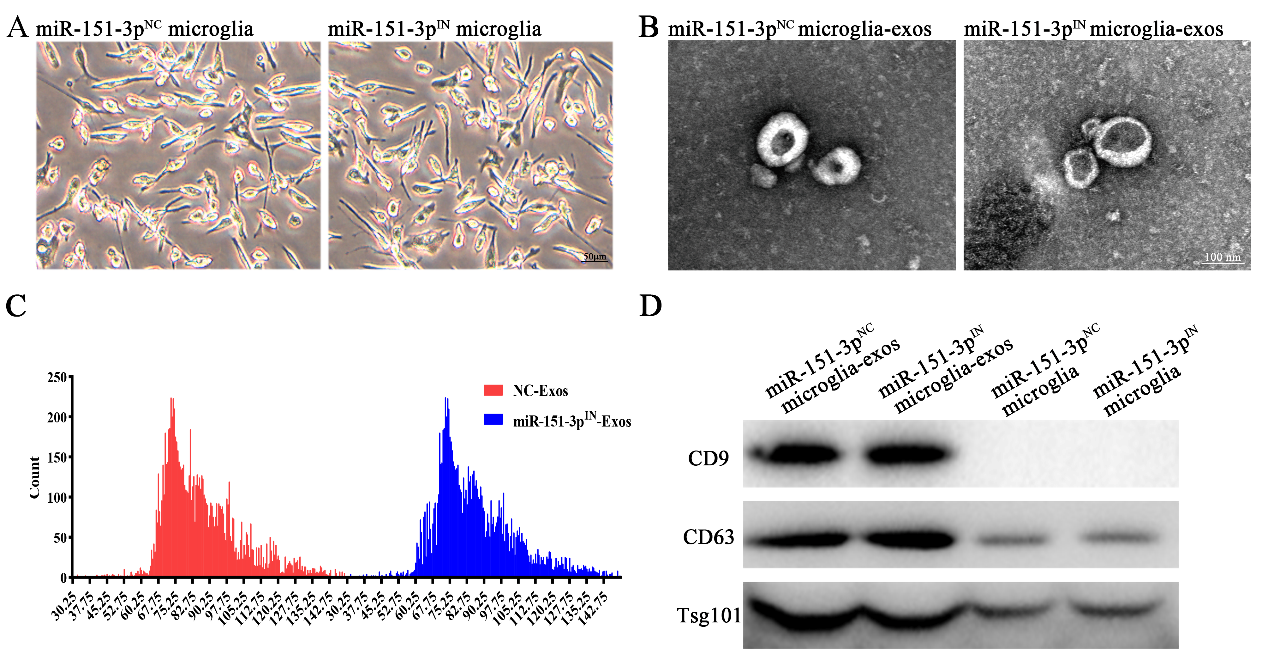


**Figure S1 Morphology and characterization of miR-151-3p^NC^ microglia and** **miR-151-3p^IN^ microglia and their exosomes.** (A) Representative microscopic image of miR-151-3p^NC^ microglia and miR-151-3p^IN^ microglia. Scale bar: 50 μm. (B) Transmission electron microscopy (TEM) image of exosomes isolated from miR-151-3p^NC^ microglia and miR-151-3p^IN^ microglia. Scale bar: 100 nm. (C) Nano-tracking analysis (NTA) showed that the diameter of microglia derived exosomes ranged from 35 to 145 nm. (D) Western blot analysis of exosome markers CD9, CD63 and Tsg101.


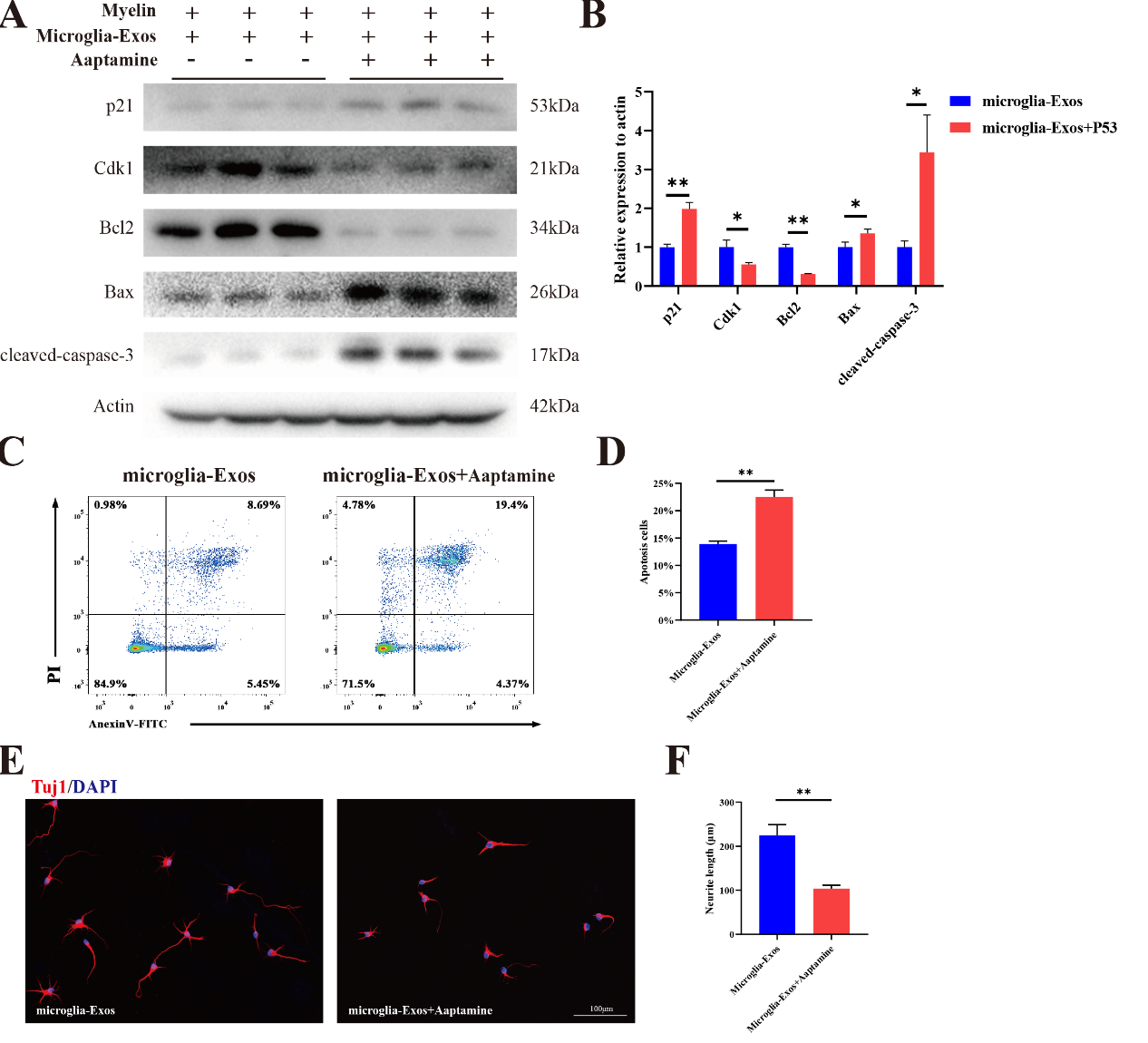


**Figure S2 Aaptamine eliminated the effect of microglia-exos on promoting axon regrowth and reducing neuron apoptosis.** (A) Western blot of p21, CDK1, apoptosis-related protein Bax and cleaved-caspase-3 and anti-apoptosis protein Bcl2 in microglia-Exos and microglia-Exos + Aaptamine treated group. (B) Quantification of relative proteins expression level in (A). N=3 per group. (C) Flow cytometry of Annexin/PI staining in microglia-Exos and microglia-Exos + Aaptamine treated group. (D) Quantification of apoptosis rate of neurons in (C). N=3 per group. (E) Representative fluorescent images of neurons staining with Tuj-1 (red) of microglia-Exos and microglia-Exos + Aaptamine treated group. Scale bar: 100 μm. (F) Quantification of neurite length in (E). N=5 per group. Data are presented as the mean ± SD. *P < 0.05, **P < 0.01.

**
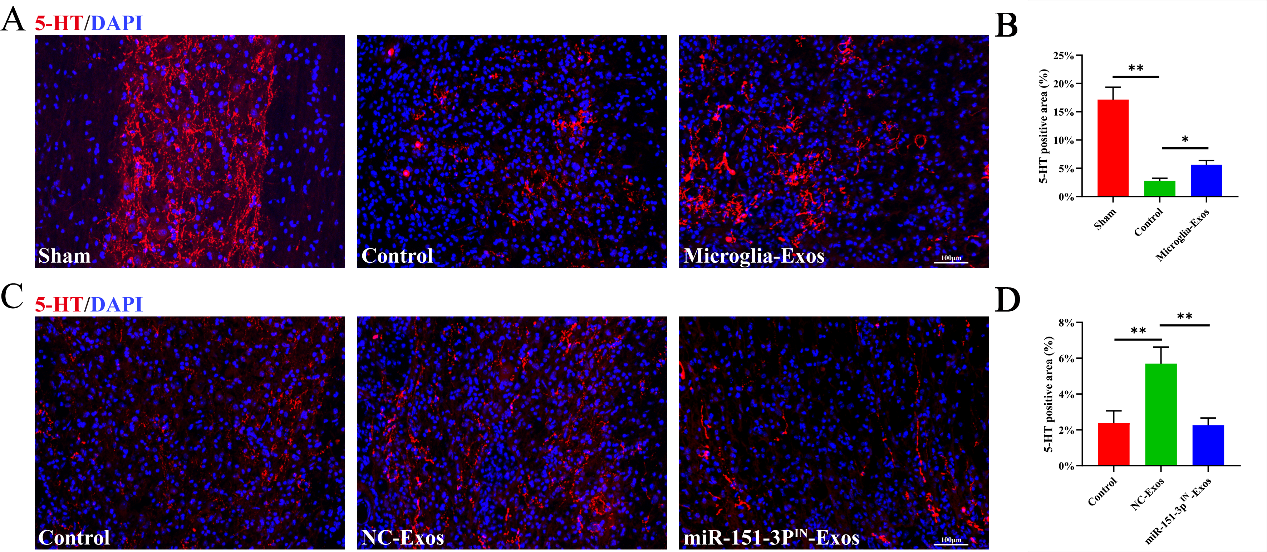
**

**Figure S3 Axon regrowth stained by 5-HT.** (A) Representative fluorescent images of neurons staining with 5-HT (red) of sham, control and Microglia-Exos treated group. Scale bar: 100 μm. (B) Quantification of 5-HT positive area (A). N=5 per group. (C) Representative fluorescent images of neurons staining with 5-HT (red) of control, microglia-Exos and miR-151-3p^IN^-Exos treated group. Scale bar: 100 μm. (D) Quantification of 5-HT positive area (C). N=5 per group. Data are presented as the mean ± SD. *P < 0.05, **P < 0.01.
